# Optimizing cardiovascular disease diagnosis through machine learning models integrating oxidized low-density lipoprotein and routine clinical indicators

**DOI:** 10.3389/fendo.2026.1850695

**Published:** 2026-07-22

**Authors:** Yilian Zhang, Jingzhu Nan, Shiyu Jiao, Zihan Liu, Hui Yuan

**Affiliations:** Department of Clinical Laboratory, Beijing Anzhen Hospital, Capital Medical University, Beijing, China

**Keywords:** cardiovascular disease, diagnostic model, machine learning, oxidized low-density lipoprotein cholesterol, XGBoost

## Abstract

**Introduction:**

To evaluate the diagnostic value of oxidized low-density lipoprotein cholesterol (oxLDL-C) for cardiovascular disease (CVD) and to construct a machine learning model integrating routine clinical indicators, thereby providing an efficient and economical tool to aid clinical diagnosis.

**Methods:**

This retrospective analysis enrolled 3, 686 participants. The discriminatory performance of oxLDL-C was compared with traditional lipid markers using receiver operating characteristic curves, and its association with CVD risk was analyzed using multivariate logistic regression. Through Recursive Feature Elimination and multivariate logistic regression, six core variables (including oxLDL-C) were ultimately selected. Seven machine learning algorithms were employed to construct predictive models, and their performance was evaluated in an internal validation set.

**Results:**

The discriminatory efficacy of oxLDL-C (AUC = 0.642) was significantly superior to traditional indicators such as low-density lipoprotein cholesterol. Its level was independently and positively associated with CVD risk (OR for the highest quartile = 3.773). The diagnostic model based on XGBoost demonstrated excellent discriminative ability (AUC = 0.911) and good calibration in internal validation.

**Discussion:**

The machine learning model integrating oxLDL-C with routine clinical indicators performs well, offering a practical tool for preliminary CVD risk screening and patient triage in resource-limited settings.

## 1 Introduction

Cardiovascular disease (CVD) represents a major global health burden (1–3), with atherosclerosis, its pathological cornerstone, being a complex process intertwined with lipid abnormalities and chronic inflammation (4–6). For a long time, low-density lipoprotein cholesterol (LDL-C) has served as the primary target for clinical CVD risk assessment and intervention due to its central role in atherogenesis (7–11).

Traditional LDL-C measurement merely reflects the quantity of this circulating lipoprotein, failing to capture its “quality” or variations in its pathogenic activity. Extensive clinical observations indicate that many patients with well-controlled LDL-C levels still experience cardiovascular events, suggesting a significant residual risk (12). The atherosclerotic potential of native LDL is truly “activated” only after its oxidative modification into oxidized low-density lipoprotein (oxLDL) (13). oxLDL-C is more readily taken up by macrophages, driving foam cell formation, and acts as a potent inflammatory activator and endothelial function disruptor, directly participating in the entire process of plaque initiation, progression, and instability. Therefore, directly measuring circulating LDL-C levels may better reflect the pro-atherogenic lipoprotein burden than measuring its precursor, LDL-C, positioning it as a promising novel diagnostic marker with greater pathological specificity.

Although the pathogenic mechanisms of oxLDL-C have been elucidated, its diagnostic value in clinical practice remains underexplored and systematically unvalidated. Existing studies often focus on mechanistic exploration or small-sample analyses, lacking systematic efficacy evaluations based on large-scale clinical cohorts. Furthermore, no study has yet integrated oxLDL-C with routine indicators to construct practical tools applicable across diverse healthcare settings. In resource-constrained primary healthcare facilities, traditional diagnostic models relying on expensive or complex investigations are often inaccessible, potentially leading to diagnostic delays. Consequently, developing an adjunct diagnostic tool based on cost-effective routine blood markers is of significant importance for optimizing healthcare resource allocation. Nowadays, machine learning algorithms are widely applied in medicine (14, 15), possessing the capability to uncover complex interrelationships and patterns within large datasets, offering new avenues for developing more precise clinical diagnostic tools (16–20). Moreover, SHAP explanation has addressed the “black box” characteristic, revealing the predictive value of each indicator for the disease (21–23).

This study aims to systematically evaluate the diagnostic efficacy of oxLDL-C for CVD using large-scale clinical data and to construct a machine learning diagnostic model integrating oxLDL-C with routine indicators. This could provide an efficient and economical preliminary screening tool for resource-limited regions. By deploying it as a web-based calculator, primary care physicians can conveniently perform risk assessments and accurately identify high-risk patients.

## 2 Materials and methods

### 2.1 Study design and population

**Figure 1:**
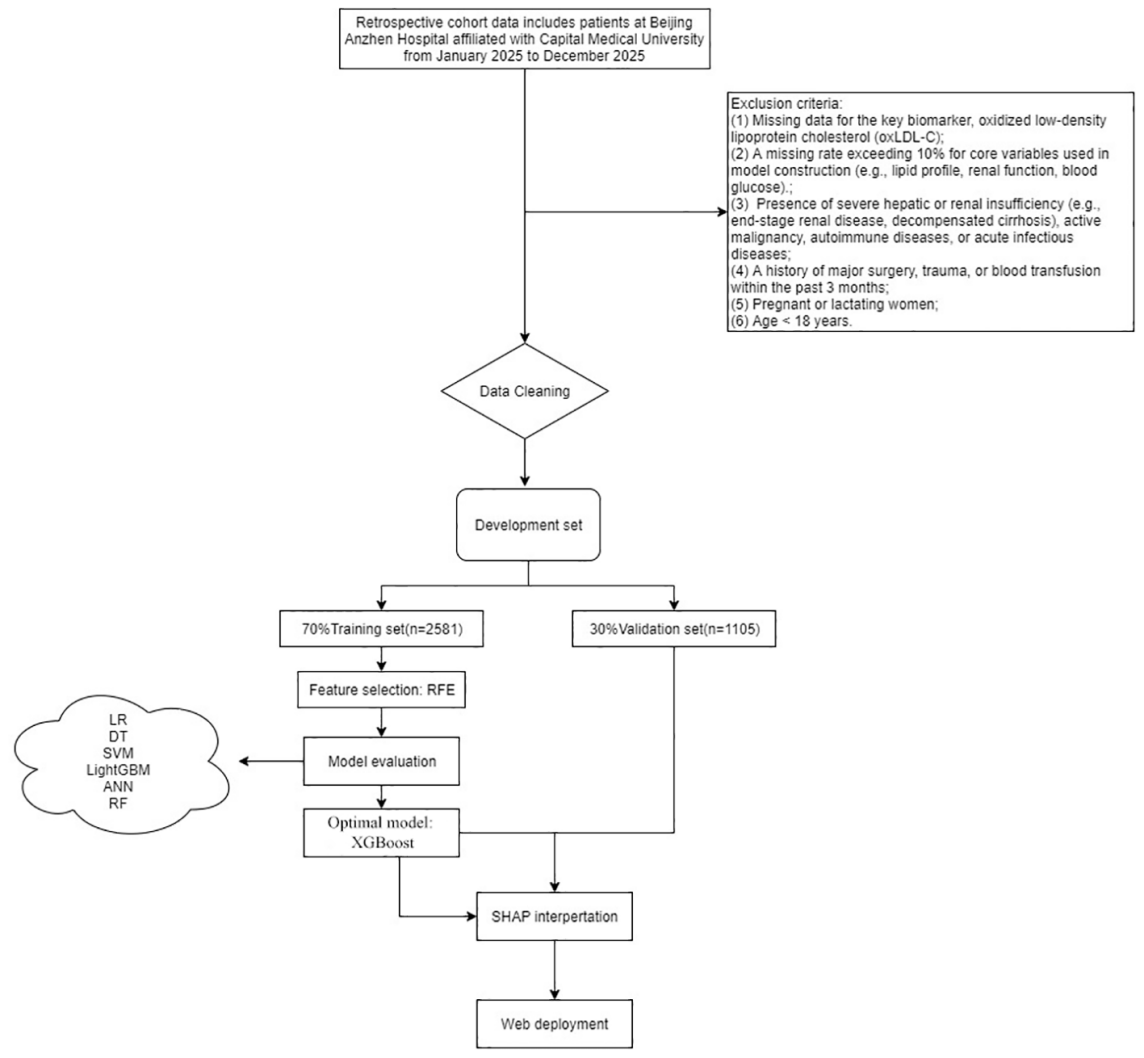
The flowchart of this study.

This study utilized data from adult patients (aged ≥18 years) who visited Beijing Anzhen Hospital, Capital Medical University, between January 1, 2025, and December 31, 2025. For the experimental group, all clinical and laboratory indicators used in the analysis were obtained from the patients’ initial examination results after a confirmed diagnosis of CVD (including heart disease or stroke). The control group consisted of individuals without any history or symptoms of CVD. The detailed inclusion and exclusion process for all study participants is shown in **Figure 1**. Ultimately, a total of 3, 686 participants were included in the analysis. This study was retrospective in design, all data were anonymized, and the requirement for informed consent from patients was waived. The study was approved by the Ethics Committee of Beijing Anzhen Hospital, Capital Medical University (ethics approval number: 2026101x).

### 2.2 Definition of cardiovascular disease

The primary outcome of this study was a definitively diagnosed cardiovascular disease (CVD), including heart disease and stroke. The diagnosis of CVD was based on the final diagnosis recorded in the electronic medical record system when the patient visited Beijing Anzhen Hospital, Capital Medical University. The diagnosis was made by clinicians after comprehensive assessment using clinical symptoms, electrocardiogram, cardiac enzymes, and imaging examinations (such as coronary angiography, echocardiography, carotid ultrasound, cranial CT, or magnetic resonance imaging). Heart disease mainly includes coronary heart disease (acute myocardial infarction, unstable angina pectoris), heart failure, and atrial fibrillation. Stroke includes ischemic stroke and hemorrhagic stroke.

### 2.3 Study variables

The primary exposure variable of interest was oxidized low-density lipoprotein cholesterol (oxLDL-C), measured using the oxidized low-density lipoprotein detection kit (immunoturbidimetric method) provided by Xi’an Goldmag Nanobiotechnology Co., Ltd., on a Beckman Coulter AU5831 clinical chemistry analyzer. Additionally, trained researchers collected demographic information, anthropometric measurements, common chronic disease histories (diabetes mellitus, hyperlipidemia), and comprehensive laboratory data, including lipid profiles, renal function, inflammatory markers, and blood cell parameters, from the hospital’s electronic medical record system. Variables with a missing rate >10% were excluded. For variables with a missing rate ≤10%, multiple imputation using the random forest algorithm in the R package ‘mice’ was performed. The proportions of missing data are presented in **Supplementary Table 1**. All imputation procedures were performed after the split of the training and validation sets: the imputation model was fitted solely on the training set and then applied to both the training and validation sets to prevent cross−dataset information leakage.

### 2.4 Statistical analysis

All data analyses were performed using R software (version 4.3.0) and Python (version 3.12). Continuous variables were described as medians (interquartile range) and compared using the Mann-Whitney U test. Categorical variables were described as frequencies (percentages) and compared using the chi-square test.

This study first compared the predictive performance of oxLDL-C and conventional lipid indicators for CVD using ROC curves. Subsequently, multivariate logistic regression models were used to evaluate the association between oxLDL-C (categorized into quartiles and as a continuous variable) and CVD risk, calculating odds ratios (OR) with 95% confidence intervals (CI). Adjustments were made sequentially for confounders including age, sex, BMI, and chronic disease history. To test the robustness of the findings, subgroup analyses stratified by demographic and clinical characteristics were performed, and restricted cubic splines (RCS) were employed to explore potential non-linear relationships between oxLDL-C and the outcome. Restricted cubic splines (RCS) with three knots were placed at the 10th, 50th, and 90th percentiles of the oxLDL−C distribution. To quantify the incremental predictive value of oxLDL−C beyond traditional clinical variables, a basic model was constructed using age, sex, BMI, history of diabetes, and history of hyperlipidemia. A full model was then built by adding oxLDL−C to the basic model. Both models were fitted on the training set, and the following incremental value metrics were evaluated in the internal validation set: ΔC−statistic, net reclassification improvement (NRI), and integrated discrimination improvement (IDI). The likelihood ratio test was used to compare the goodness−of−fit between the two models.

Subsequently, the overall sample was randomly divided into a training set and an internal validation set at a ratio of 7:3. To construct the prediction models, a systematic feature selection process was performed. To reduce redundancy among features and enhance model stability, a hybrid feature selection strategy combining filter and wrapper methods was adopted. First, highly correlated variables (|r| ≥ 0.7, FDR−p < 0.05) were removed using Pearson correlation analysis, leaving relatively independent variables. The rationale of this filtering step aligns with the “minimum redundancy” criterion of the mRMR algorithm, aiming to minimize information overlap among the selected features. Subsequently, recursive feature elimination (RFE) was applied on the set of independent variables to identify core predictors, which were then intersected with the variables selected by multivariable logistic regression. This intersection approach effectively reduces dependence on a single model (the base model of RFE). Seven machine learning models (e.g., logistic regression, support vector machine, random forest, XGBoost, etc.) were built and compared on the training set. The optimal model was selected by evaluating its discriminative performance (AUC), calibration, and clinical net benefit on the validation set. In addition to the above metrics, we also calculated the Matthews correlation coefficient (MCC) to provide a less biased estimate of the classifier’s predictive ability. Finally, the SHAP framework was used to interpret the decision logic of the optimal model, and a web-based calculator was deployed to facilitate clinical use. All feature selection steps (correlation filtering, recursive feature elimination [RFE]) were completed solely based on the training set data; the validation set was used only for final model evaluation and did not participate in any variable selection process.

## 3 Results

### 3.1 Baseline characteristics

**Table 1** shows that a total of 3, 686 participants were included in this study, of whom 1, 925 (52.22%) had CVD. Patients in the CVD group were older (median 61.0 years vs. 52.0 years), had a higher proportion of males (66.81% vs. 51.22%), and a higher prevalence of diabetes mellitus (68.57% vs. 42.25%), with all differences being statistically significant (all P < 0.001). The core indicator, oxLDL-C level, was significantly higher in the CVD group compared to the non-CVD group (median 43.29 U/L vs. 34.47 U/L, P < 0.001). Furthermore, significant differences were observed between the two groups in BMI, renal function (eGFR), lipid profile (HDL-C), inflammatory markers (hs-CRP, NLR), and various blood cell parameters (all P < 0.05). The prevalence of hyperlipidemia did not differ significantly between the groups.

**Table 1:** Baseline characteristics of the overall study population.

Variables	Total(n=3686)	Without CVD(n=1761)	With CVD(n=1925)	p
Age	57.00 [46.00, 66.00]	52.00 [42.00, 62.00]	61.00 [52.00, 68.00]	<0.001
BMI	24.90 [22.70, 27.00]	25.10 [23.10, 27.20]	24.60 [22.50, 26.70]	<0.001
Sex				<0.001
Male	2188 (59.36)	902 (51.22)	1286 (66.81)	
Female	1498 (40.64)	859 (48.78)	639 (33.19)	
DM	2064 (56.00)	744 (42.25)	1320 (68.57)	<0.001
Hyperlipidemia	963 (26.13)	460 (26.12)	503 (26.13)	1
stroke	563 (15.27)	0 (0.00)	563 (29.25)	<0.001
heart disease	1895 (51.41)	0 (0.00)	1895 (98.44)	<0.001
oxLDL-C(U/L)	39.28 [27.90, 52.03]	34.47 [24.48, 46.05]	43.29 [32.40, 56.30]	<0.001
GGT(U/L)	23.00 [15.00, 36.00]	20.00 [13.00, 33.00]	25.00 [17.00, 39.00]	<0.001
ALB(g/dl)	4.54 [4.31, 4.75]	4.61 [4.44, 4.80]	4.46 [4.17, 4.70]	<0.001
ALT(U/L)	19.00 [13.00, 29.00]	18.00 [12.00, 26.00]	20.30 [14.00, 30.80]	<0.001
hs-CRP(mg/L)	0.86 [0.48, 1.95]	0.78 [0.46, 1.61]	0.95 [0.50, 2.38]	<0.001
ChE(kU/L)	9.00 [7.80, 10.10]	9.20 [8.10, 10.30]	8.70 [7.60, 9.90]	<0.001
LDL-C(mmol/L)	3.07 [2.48, 3.63]	3.04 [2.48, 3.59]	3.09 [2.48, 3.67]	0.093
TG(mmol/L)	1.30 [0.94, 1.83]	1.29 [0.92, 1.87]	1.31 [0.96, 1.81]	0.588
HDL-C(mmol/L)	1.21 [1.02, 1.47]	1.34 [1.12, 1.60]	1.12 [0.95, 1.34]	<0.001
CK(U/L)	83.95 [62.00, 117.00]	86.00 [65.00, 118.00]	82.00 [59.00, 114.00]	<0.001
CK-MB(ng/ml)	1.50 [1.10, 2.00]	1.40 [1.10, 1.80]	1.70 [1.20, 2.30]	<0.001
ALP(U/L)	73.00 [60.00, 87.00]	69.00 [57.00, 83.00]	76.00 [64.00, 91.00]	<0.001
UA(umol/L)	320.10 [263.05, 380.20]	319.30 [262.80, 384.20]	320.90 [263.20, 378.50]	0.952
Glu(mmol/L)	5.39 [4.95, 6.12]	5.19 [4.86, 5.65]	5.67 [5.10, 6.63]	<0.001
PA (g/L)	0.26 [0.23, 0.30]	0.27 [0.24, 0.30]	0.26 [0.22, 0.29]	<0.001
LD (U/L)	172.00 [154.00, 193.00]	167.00 [150.00, 185.00]	177.50 [158.00, 201.00]	<0.001
GA (g/dl)	0.54 [0.49, 0.61]	0.53 [0.49, 0.58]	0.55 [0.49, 0.64]	<0.001
AST(U/L)	19.00 [16.00, 24.00]	18.00 [16.00, 23.00]	20.00 [17.00, 26.00]	<0.001
HCY(umol/L)	11.40 [9.30, 14.30]	11.00 [9.10, 13.60]	12.00 [9.50, 15.10]	<0.001
DB(umol/L)	4.09 [3.14, 5.34]	3.83 [3.02, 4.83]	4.40 [3.30, 5.89]	<0.001
CHO(mmol/L)	4.38 [3.48, 5.25]	5.09 [4.45, 5.71]	3.58 [3.08, 4.37]	<0.001
TB(umol/L)	11.23 [8.46, 15.13]	10.30 [7.72, 13.72]	12.26 [9.35, 16.33]	<0.001
eGFR(mL/min/1.73m^2)	93.45 [80.40, 103.31]	100.21 [90.39, 108.87]	86.79 [72.95, 96.78]	<0.001
AG-Ratio	1.66 [1.50, 1.83]	1.62 [1.48, 1.78]	1.70 [1.53, 1.88]	<0.001
AG(mmol/L)	16.30 [14.30, 17.90]	16.50 [14.80, 18.10]	16.10 [13.50, 17.70]	<0.001
TBA(umol/L)	3.10 [2.10, 4.80]	3.00 [2.00, 4.50]	3.20 [2.10, 5.10]	0.004
WBC(×10^9/L)	6.54 [5.47, 7.81]	6.37 [5.38, 7.60]	6.67 [5.62, 8.06]	<0.001
RBC(×10^12/L)	4.81 [4.43, 5.16]	4.87 [4.51, 5.21]	4.74 [4.34, 5.11]	<0.001
Hb(g/L)	147.00 [134.00, 157.75]	148.00 [136.00, 158.00]	145.00 [132.00, 156.00]	<0.001
HCT	43.20 [39.90, 46.30]	43.70 [40.40, 46.50]	42.90 [39.50, 46.00]	<0.001
MCV(fL)	89.95 [87.60, 92.60]	89.60 [87.20, 92.10]	90.30 [88.00, 93.00]	<0.001
MCH(pg)	30.50 [29.50, 31.40]	30.40 [29.40, 31.30]	30.60 [29.60, 31.60]	<0.001
MCHC(g/L)	338.00 [333.00, 343.00]	339.00 [333.00, 344.00]	338.00 [332.00, 343.00]	0.002
PLT(×10^9/L)	219.00 [183.00, 259.00]	232.00 [198.00, 272.00]	208.00 [173.00, 247.00]	<0.001
MPV(fL)	9.50 [8.90, 10.30]	9.50 [8.90, 10.20]	9.60 [8.90, 10.30]	0.509
NEUT(×10^9/L)	3.99 [3.18, 5.10]	3.83 [3.06, 4.76]	4.20 [3.32, 5.37]	<0.001
LYM(×10^9/L)	1.82 [1.43, 2.26]	1.89 [1.52, 2.33]	1.77 [1.35, 2.19]	<0.001
MONO(×10^9/L)	0.38 [0.30, 0.47]	0.36 [0.29, 0.45]	0.40 [0.31, 0.49]	<0.001
EOS(×10^9/L)	0.11 [0.07, 0.19]	0.12 [0.07, 0.19]	0.11 [0.06, 0.19]	0.043
BAS(×10^9/L)	0.02 [0.02, 0.04]	0.03 [0.02, 0.04]	0.02 [0.02, 0.03]	<0.001
PCT	0.21 [0.18, 0.25]	0.22 [0.19, 0.26]	0.20 [0.17, 0.23]	<0.001
PDW	16.10 [15.90, 16.40]	16.10 [15.90, 16.40]	16.20 [15.90, 16.40]	0.353
RDW-SD(fl)	41.90 [40.40, 43.58]	41.50 [40.10, 43.00]	42.20 [40.70, 44.00]	<0.001
RDW-CV	12.90 [12.60, 13.40]	12.90 [12.60, 13.30]	13.00 [12.60, 13.50]	<0.001
P-LCR	22.80 [18.40, 28.00]	22.60 [18.60, 27.90]	23.10 [18.20, 28.20]	0.524
NT-proBNP(pg/mL)	105.28 [40.04, 449.50]	79.00 [31.00, 247.78]	154.00 [53.00, 769.46]	<0.001
NLR	2.15 [1.64, 2.95]	2.00 [1.56, 2.67]	2.32 [1.75, 3.32]	<0.001
PLR	120.92 [94.42, 157.29]	122.75 [97.56, 154.84]	119.01 [91.52, 158.96]	0.047
TyG	8.68 [8.31, 9.07]	8.60 [8.23, 9.03]	8.73 [8.39, 9.09]	<0.001
CHG	5.15 [4.91, 5.42]	5.17 [4.93, 5.44]	5.13 [4.88, 5.40]	<0.001
PHR	176.59 [139.56, 228.87]	171.92 [134.92, 220.80]	182.11 [143.33, 235.92]	<0.001
dNLR	0.88 [0.85, 0.91]	0.88 [0.85, 0.90]	0.88 [0.86, 0.91]	0.002
MLR	0.20 [0.16, 0.27]	0.19 [0.15, 0.24]	0.22 [0.17, 0.30]	<0.001
NMLR	2.35 [1.81, 3.21]	2.20 [1.71, 2.90]	2.55 [1.93, 3.61]	<0.001
SIRI	0.81 [0.56, 1.24]	0.73 [0.51, 1.05]	0.91 [0.61, 1.45]	<0.001
SII	471.52 [341.80, 694.07]	465.00 [340.47, 655.71]	484.54 [342.06, 738.09]	0.001

BMI, body mass index; CVD, cardiovascular disease; DM, diabetes mellitus; oxLDL-C, oxidized low-density lipoprotein cholesterol; GGT, gamma-glutamyl transferase; ALB, albumin; ALT, alanine aminotransferase; hs-CRP, high-sensitivity C-reactive protein; ChE, cholinesterase; LDL-C, low-density lipoprotein cholesterol; TG, triglycerides; HDL-C, high-density lipoprotein cholesterol; CK, creatine kinase; CK-MB, creatine kinase-MB; ALP, alkaline phosphatase; UA, uric acid; Glu, glucose; PA, prealbumin; LD, lactate dehydrogenase; GA, glycated albumin; AST, aspartate aminotransferase; HCY, homocysteine; DB, direct bilirubin; CHO, total cholesterol; TB, total bilirubin; eGFR, estimated glomerular filtration rate; AG, anion gap; AG-Ratio, albumin-to-globulin ratio; TBA, total bile acids; WBC, white blood cell count; RBC, red blood cell count; Hb, hemoglobin; HCT, hematocrit; MCV, mean corpuscular volume; MCH, mean corpuscular hemoglobin; MCHC, mean corpuscular hemoglobin concentration; PLT, platelet count; MPV, mean platelet volume; NEUT, neutrophils; LYM, lymphocytes; MON, monocytes; EOS, eosinophils; BAS, basophils; PCT, procalcitonin; PDW, platelet distribution width; RDW-SD, red cell distribution width standard deviation; RDW-CV, red cell distribution width coefficient of variation; P-LCR, platelet larger cell ratio; NT-proBNP, N-terminal pro-brain natriuretic peptide; NLR, neutrophil-to-lymphocyte ratio; PLR, platelet-to-lymphocyte ratio; TyG, triglyceride-glucose index; CHG, cholesterol-glucose index; PHR, platelet-to-hemoglobin ratio; dNLR, derived neutrophil-to-lymphocyte ratio; MLR, monocyte-to-lymphocyte ratio; NMLR, neutrophil-monocyte-to-lymphocyte ratio; SIRI, systemic inflammation response index; SII, systemic immune-inflammation index.

All participants were randomly divided into a training set (n = 2581) and an internal validation set (n = 1105) in a 7:3 ratio. The baseline characteristics of the training set and internal validation set are presented in **Supplementary Tables 2**, **3**, respectively, showing balanced distributions of key characteristics between the two groups.

### 3.2 Diagnostic value of oxLDL-C and other indicators for cardiovascular disease

**Figure 2:**
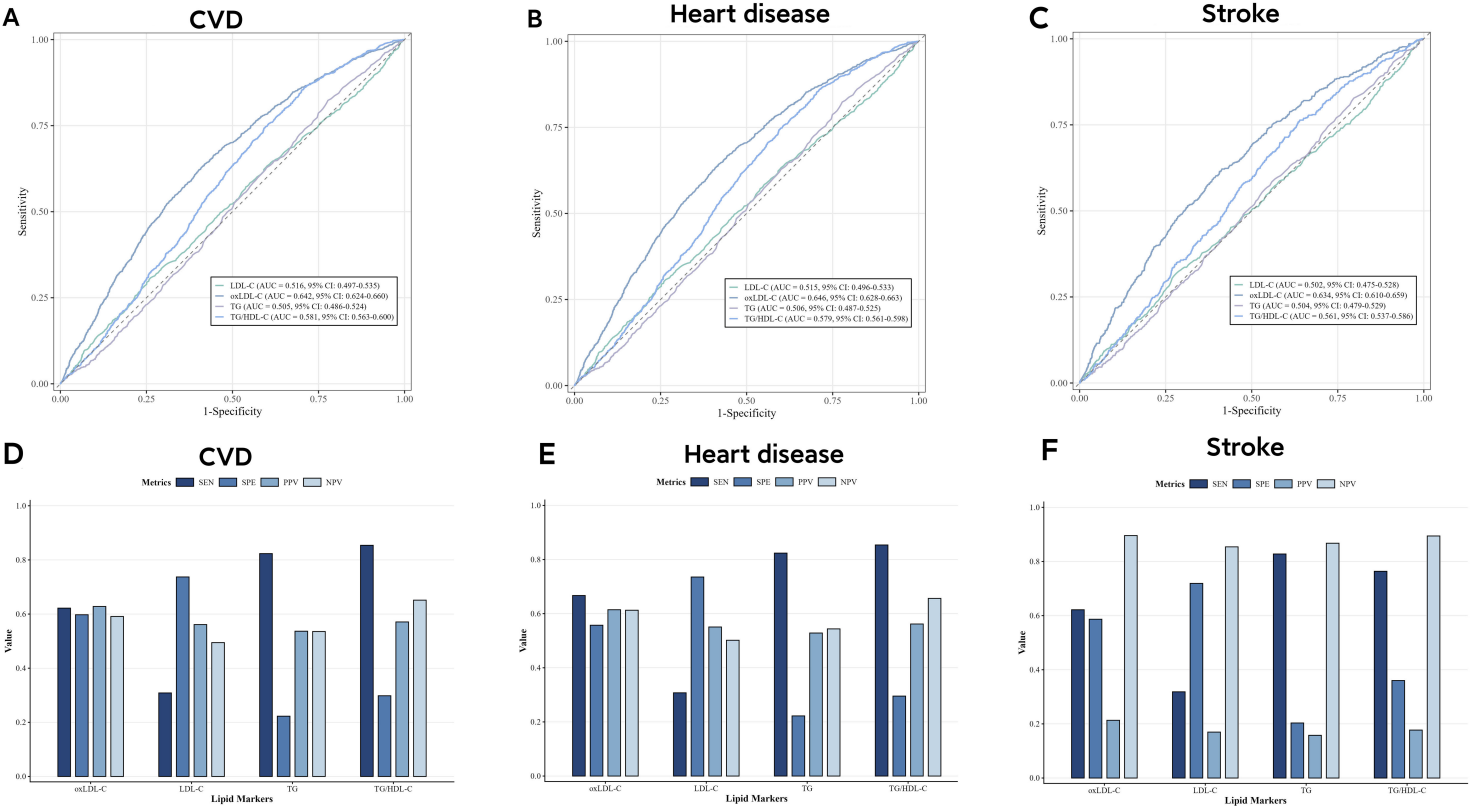
Performance comparison of oxLDL-C and conventional lipid indicators in predicting the risk of cardiovascular diseases. **(A-C)** Receiver operating characteristic (ROC) curves of oxidized low-density lipoprotein cholesterol (oxLDL-C), low-density lipoprotein cholesterol (LDL-C), triglycerides (TG), and the triglyceride to high-density lipoprotein cholesterol ratio (TG/HDL-C) for predicting **(A)** overall cardiovascular disease (CVD), **(B)** heart disease, and **(C)** stroke, respectively. **(D–F)** Corresponding performance metrics at the optimal cutoff values—sensitivity (SEN), specificity (SPE), positive predictive value (PPV), and negative predictive value (NPV)—for each predictor regarding the three disease outcomes mentioned above. oxLDL-C, oxidized low-density lipoprotein cholesterol; LDL-C, low-density lipoprotein cholesterol; TG, triglycerides; HDL-C, high-density lipoprotein cholesterol; CVD, cardiovascular disease; ROC, receiver operating characteristic curve; AUC, area under the curve; SEN, sensitivity; SPE, specificity; PPV, positive predictive value; NPV, negative predictive value.

To evaluate the predictive performance of oxLDL-C for CVD, we compared it with traditional lipid markers including LDL-C, triglycerides (TG), and the triglyceride-to-high-density lipoprotein cholesterol ratio (TG/HDL-C). The results are shown in **Figure 2**.

For assessing overall CVD risk, the AUC for oxLDL-C was 0.642 (95% CI: 0.624--0.660), which was significantly higher than that for LDL-C (AUC: 0.516), TG (AUC: 0.505), and TG/HDL-C (AUC: 0.581). For specific types of cardiovascular events, oxLDL-C also demonstrated the best discriminative ability: the AUC for predicting heart disease was 0.646 (95% CI: 0.628--0.663), and for predicting stroke, it was 0.634 (95% CI: 0.610--0.659). The optimal cut-off values for oxLDL-C discriminating CVD, heart disease, and stroke were 38.495 U/L, 41.595 U/L, and 36.790 U/L, respectively. In all comparisons, the AUC values for oxLDL-C were significantly superior to those of the other three indicators (all P < 0.05). Furthermore, the optimal cut-off values determined by the Youden index provide potential reference points for clinical application.

These results indicate that oxLDL-C has superior predictive performance for discriminating CVD, heart disease, and stroke compared to traditional markers like LDL-C, TG, and TG/HDL-C.

### 3.3 Association analysis between oxLDL-C and cardiovascular disease risk

Multivariate logistic regression models were used to evaluate the association between oxLDL-C levels and the risk of CVD, heart disease, and stroke. As shown in **Table 2**, oxLDL-C levels were significantly positively associated with the risk of all three outcomes, and this association remained robust after stepwise adjustment for confounding factors.

**Table 2:** Multivariable regression analysis of the associations between oxLDL-C and cardiovascular diseases.

oxLDL-C	Model1		Model2		Model3	
	OR(95%CI)	P.value	OR(95%CI)	P.value	OR(95%CI)	P.value
CVD						
Q1	reference		reference		reference	
Q2	1.654(1.372-1.994)	<0.001	1.736(1.421-2.123)	<0.001	1.727(1.406-2.124)	<0.001
Q3	2.580(2.139-3.116)	<0.001	2.732(2.233-3.348)	<0.001	2.704(2.198-3.331)	<0.001
Q4	3.737(3.085-4.535)	<0.001	3.814(3.105-4.694)	<0.001	3.773(3.055-4.670)	<0.001
PerSD	1.721(1.574-1.886)	<0.001	1.706(1.553-1.879)	<0.001	1.689(1.533-1.864)	<0.001
Heart disease						
Q1	reference		reference		reference	
Q2	1.722(1.428-2.079)	<0.001	1.807(1.479-2.209)	<0.001	1.799(1.466-2.210)	<0.001
Q3	2.697(2.234-3.260)	<0.001	2.839(2.322-3.477)	<0.001	2.810(2.288-3.458)	<0.001
Q4	3.898(3.217-4.733)	<0.001	3.964(3.230-4.876)	<0.001	3.910(3.171-4.832)	<0.001
PerSD	1.726(1.579-1.891)	<0.001	1.708(1.556-1.880)	<0.001	1.688(1.535-1.861)	<0.001
Stroke						
Q1	reference		reference		reference	
Q2	1.789(1.321-2.437)	<0.001	1.863(1.362-2.564)	<0.001	1.875(1.370-2.582)	<0.001
Q3	2.145(1.598-2.903)	<0.001	2.265(1.668-3.099)	<0.001	2.288(1.684-3.132)	<0.001
Q4	3.768(2.855-5.025)	<0.001	3.715(2.782-5.010)	<0.001	3.791(2.835-5.119)	<0.001
PerSD	1.490(1.357-1.638)	<0.001	1.461(1.323-1.616)	<0.001	1.472(1.331-1.629)	<0.001

Model 1 was unadjusted. Model 2 was adjusted for age, sex, and BMI. Model 3 was additionally adjusted for diabetes and hyperlipidemia. P for trend <0.001 for all outcomes.

CVD, cardiovascular disease; OR, odds ratio; CI, confidence interval; SD, standard deviation.

In Model 3, compared to individuals in the lowest oxLDL-C quartile (Q1), those in the highest quartile (Q4) had a significantly increased risk of CVD (OR = 3.773, 95% CI: 3.055--4.670), heart disease (OR = 3.910, 95% CI: 3.171--4.832), and stroke (OR = 3.791, 95% CI: 2.835--5.119), respectively. When oxLDL-C was analyzed as a continuous variable, each one-standard deviation (SD) increase was associated with a 69% increased risk of CVD (OR = 1.689, 95% CI: 1.533--1.864), a 69% increased risk of heart disease (OR = 1.688, 95% CI: 1.535--1.861), and a 47% increased risk of stroke (OR = 1.472, 95% CI: 1.331--1.629). All test results were statistically significant (P < 0.001).

### 3.4 Incremental predictive value of oxLDL−C

In the validation set of **Table 3**, the basic model (including Age, Sex, BMI, DM, and Hyperlipidemia) achieved a C−statistic of 0.729 (95% CI: 0.697–0.759), with statistical significance (P < 0.001), indicating good discriminative ability. After adding oxLDL−C to the basic model, the C−statistic of the full model increased to 0.755 (95% CI: 0.727–0.782), also statistically significant (P < 0.001).

**Table 3:** Incremental value of oxLDL-C over the basic model for CVD diagnosis in the validation set.

Model	C Statistic Estimate (95% CI)	C Statistic P value	NRI Estimate (95% CI)	NRI P value	IDI Estimate (95% CI)	IDI P value	LR χ²	LR P value
Basic model	0.729 (0.697-0.759)	<0.001	Reference		Reference		Reference	
Basic model + oxLDL-C	0.755 (0.727-0.782)	<0.001	0.4691 (0.3526-0.5766)	<0.001	0.0320 (0.0235-0.0403)	<0.001	72.534	<0.001

The basic model includes age, sex, body mass index (BMI), diabetes mellitus (DM), and hyperlipidemia.

NRI, net reclassification improvement; IDI, integrated discrimination improvement; LR χ², likelihood ratio chi-square statistic; CI, confidence interval; oxLDL-C, oxidized low-density lipoprotein cholesterol; CVD, cardiovascular disease.

All metrics are derived from the internal validation set (n = 1, 105). The NRI and IDI quantify the incremental predictive value of adding oxLDL-C to the basic model. The likelihood ratio test compares the goodness-of-fit between the basic model and the basic model plus oxLDL-C.

Further evaluation of the incremental predictive value of oxLDL−C showed a net reclassification improvement (NRI) of 0.4691 (95% CI: 0.3526–0.5766, P < 0.001), indicating that adding oxLDL−C to the basic model improved net reclassification by 46.91%. The integrated discrimination improvement (IDI) was 0.0320 (95% CI: 0.0235–0.0403, P < 0.001), suggesting a statistically significant improvement in overall discrimination. The likelihood ratio test yielded a χ² of 72.534 (P < 0.001), further confirming that adding oxLDL−C significantly improved model fit.

In summary, adding oxLDL−C to the basic model (Age, Sex, BMI, DM, Hyperlipidemia) significantly enhances predictive performance, indicating that oxLDL−C provides independent incremental predictive value.

### 3.5 Association of oxLDL-C with outcomes across different populations

**Figure 3:**
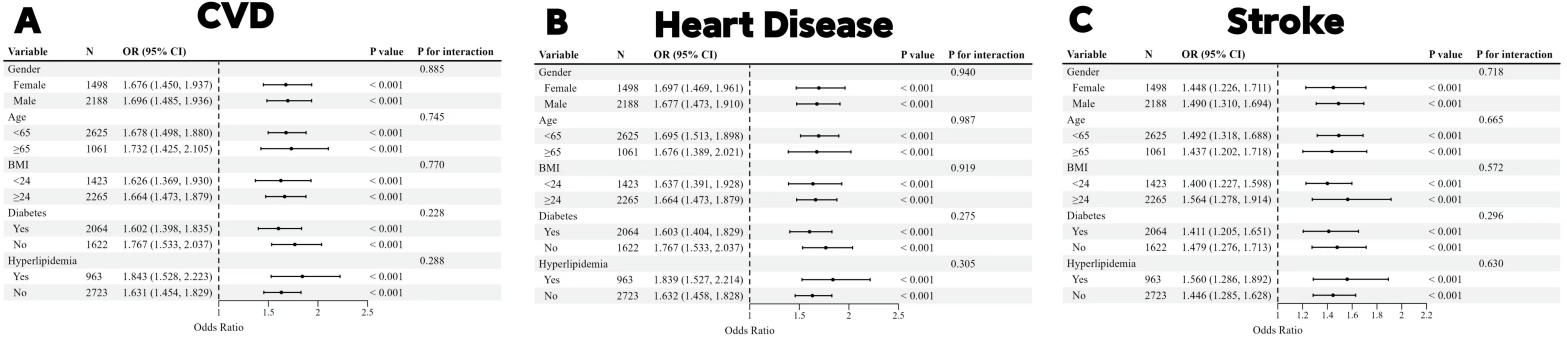
Subgroup analyses of the association between oxLDL-C and the risk of cardiovascular outcomes. Notes: Forest plots present the odds ratios (ORs) and 95% confidence intervals for per standard deviation increase in oxLDL-C in relation to the risk of **(A)** cardiovascular disease (CVD), **(B)** heart disease, and **(C)** stroke, stratified by gender, age, BMI, diabetes, and hyperlipidemia status. All models were adjusted for age, sex, BMI, diabetes, and hyperlipidemia. P for interaction represents the P-value for interaction. Abbreviations: oxLDL-C, oxidized low-density lipoprotein cholesterol; CVD, cardiovascular disease; OR, odds ratio; CI, confidence interval; BMI, body mass index.

The association between oxLDL-C and CVD risk remained generally robust across most subgroups. After stratification by sex, age, BMI, diabetes mellitus, and hyperlipidemia status, each one-SD increase in oxLDL-C was significantly associated with increased risk of CVD and heart disease in all subgroups (all P < 0.05). For stroke, a significant positive association was also observed in all other subgroups (all P < 0.001). Interaction analysis revealed no significant interactions across any of the pre-specified subgroups (all P for interaction > 0.05) (**Figure 3**).

### 3.6 Non-linear association between oxLDL-C and cardiovascular disease risk

**Figure 4:**
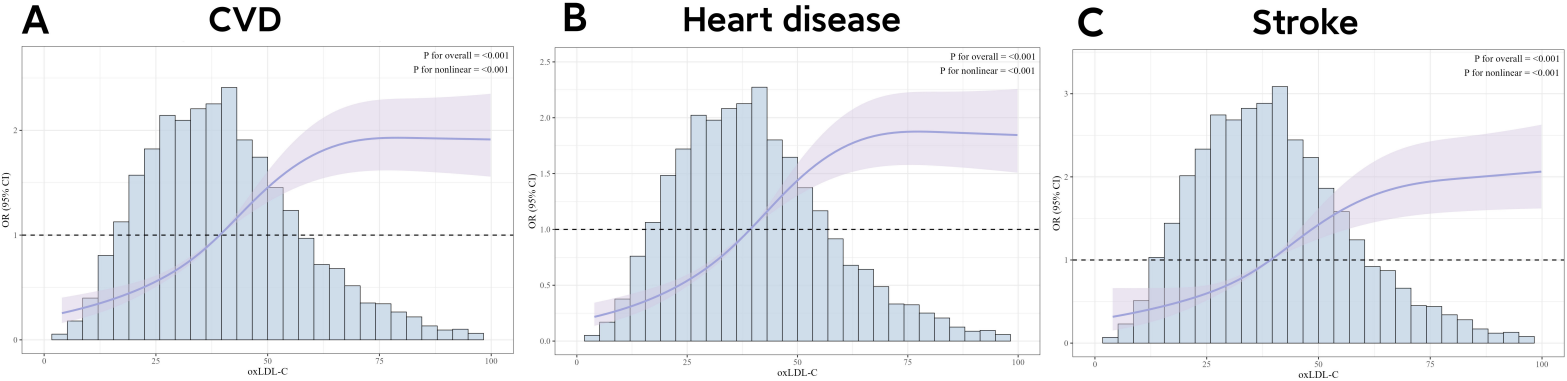
Dose-response curves depicting the association between oxLDL-C and cardiovascular disease risk. **(A)** Relationship between oxLDL-C and total cardiovascular disease (CVD). **(B)** Relationship between oxLDL-C and heart disease.**(C)** Relationship between oxLDL-C and stroke. Curves were fitted using restricted cubic splines. All models were adjusted for age, sex, body mass index (BMI), diabetes, and hyperlipidemia. The solid line represents the odds ratio, and the shaded area denotes the 95% confidence interval. The dashed reference line is set at a odds ratio of 1.0. Both the tests for overall association and nonlinearity were statistically significant (P < 0.001 for all endpoints).oxLDL−C, oxidized low−density lipoprotein cholesterol; CVD, cardiovascular disease; BMI, body mass index.

To explore potential non-linear associations between oxLDL-C and cardiovascular outcomes, RCS analysis was performed for dose-response analysis (**Figure 4**). RCS with three knots were placed at the 10th, 50th, and 90th percentiles of the oxLDL−C distribution. The results revealed a strong non-linear dose-response relationship between oxLDL-C levels and the risk of overall CVD, heart disease, and stroke (all P for overall and P for nonlinear < 0.001). These curves indicate that the risk of cardiovascular events does not increase simply linearly with rising oxLDL-C levels, but rather changes more markedly within specific intervals.

### 3.7 Feature selection

**Figure 5:**
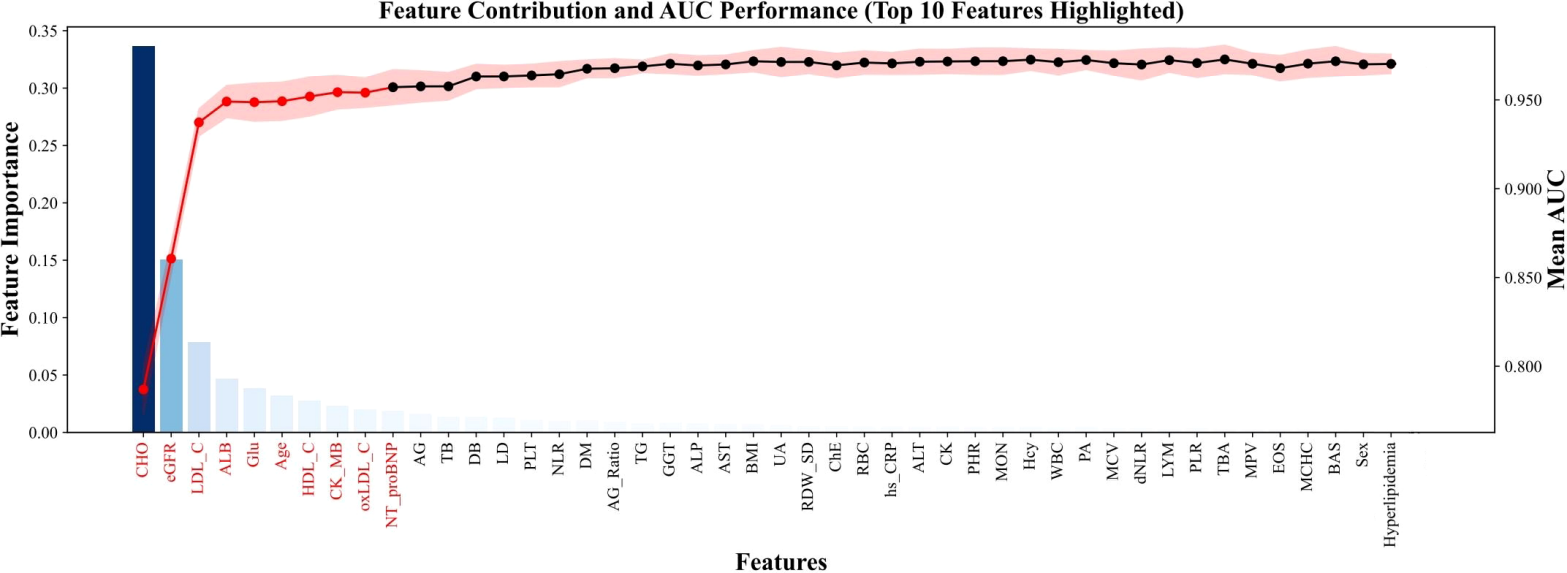
Recursive feature elimination (RFE) process and feature contribution. Auc, Area Under the Curve; CHO, Cholesterol; eGFR, estimated Glomerular Filtration Rate; LDL-C, Low-Density Lipoprotein Cholesterol; ALB, Albumin; HDL-C, High-Density Lipoprotein Cholesterol; CK-MB, Creatine Kinase-MB; oxLDL-C, oxidized low-density lipoprotein cholesterol; NT-proBNP, N-terminal pro-B-type Natriuretic Peptide; AG, anion gap; TB, total bilirubin; DB, direct bilirubin; LD, lactate dehydrogenase; PLT, platelet count; NLR, neutrophil-to-lymphocyte ratio; DM, diabetes mellitus; AG-Ratio, Albumin-to-globulin ratio; TG, triglycerides; GGT, gamma-glutamyl transferase; ALP, alkaline phosphatase; AST, aspartate aminotransferase; BMI, body mass index; UA, uric acid; RDW-SD, red cell distribution width standard deviation; ChE, cholinesterase; RBC, red blood cell count; hs-CRP, high-sensitivity C-reactive protein; ALT, alanine aminotransferase; CK, creatine kinase; PHR, platelet-to-hemoglobin ratio; MON, Monocyte;HCY, homocysteine; WBC, white blood cell count; PA, prealbumin; MCV, mean corpuscular volume; dNLR, derived neutrophil-to-lymphocyte ratio; LYM, lymphocytes; PLR, platelet-to-lymphocyte ratio; TBA, total bile acids; MPV, mean platelet volume; EOS, eosinophils; MCHC, mean corpuscular hemoglobin concentration; BAS, basophils; RFE, Recursive Feature Elimination.

This study employed a multi-step feature selection method to identify core predictors from the 62 initial variables, performed in the training set. Multivariate logistic regression and Recursive Feature Elimination (RFE) were used for selection: multivariate logistic regression was applied to variables with P < 0.05 in the baseline table of the training set, yielding 24 variables; based on correlation analysis, for every two highly correlated variables, the variable with greater value for the outcome is retained based on clinical experience, resulting in the retention of 47 relatively independent variables, from which RFE selected the top 8 variables. The intersection of variables from multivariate logistic regression and RFE contained six core variables: total cholesterol (CHO), estimated glomerular filtration rate (eGFR), glucose (Glu), high-density lipoprotein cholesterol (HDL-C), creatine kinase-MB (CK-MB), and oxidized low-density lipoprotein cholesterol (oxLDL-C). Collinearity diagnostics for these six variables showed variance inflation factors (VIF) all below 5, indicating no significant multicollinearity. The correlation analysis heatmap is shown in **Supplementary Figure 1**. RFE variable selection is illustrated in **Figure 5** (RFE feature importance ranking), multivariate logistic regression results are in **Supplementary Table 4**, and collinearity diagnostics are in **Supplementary Table 5**. To evaluate the stability of feature selection via recursive feature elimination (RFE), nested cross-validation was employed (outer 10-fold × inner 5-fold), with RFE independently executed within each outer fold to select the top 10 features. The results showed that the outer-fold test AUC ranged from 0.894 to 0.950, with minimal fluctuation across folds. Nine variables — Age, oxLDL-C, ALB, LDL-C, HDL-C, CK-MB, Glu, CHO, and eGFR — were consistently selected across all 10 folds, with only the terminal feature alternating between AG and NT-proBNP (**Supplementary Table 6**). The coefficient of variation of feature selection frequency was 0.267 (< 0.3), indicating satisfactory stability of the RFE feature selection process and demonstrating that the selected feature set is not dependent on a specific data partition.

### 3.8 Machine learning model evaluation and comparison

Based on the selected features, seven machine learning models were constructed and systematically evaluated in the training set. As shown in **Table 4**, the XGBoost model demonstrated excellent discriminative performance (AUROC = 0.942, 95% CI: 0.933–0.950) with balanced accuracy (0.872), precision (0.882), and sensitivity (0.872) in the training set. The five-fold cross-validation results showed a mean AUC of 0.911 with a standard deviation of 0.009. This low standard deviation indicates excellent model stability, meaning that the model’s discriminative performance is insensitive to different partitions of the training data. In the internal validation set, XGBoost maintained good generalizability (AUROC = 0.911, 95% CI: 0.893–0.927), outperforming traditional models such as logistic regression, decision tree, and support vector machine, and exhibited high stability. The MCC values for all models on both the training and validation sets are presented in **Table 4**. Notably, the MCC of XGBoost on the validation set was 0.676, indicating good consistency in predicting both positive and negative classes.

**Table 4:** Performance evaluation of machine learning models on the training set and internal validation set.

Model	AUROC (95% CI)	Accuracy	Precision	Sensitivity	Specificity	F1 Score	Kappa	Youden’s J	PPV	NPV	MCC
Training set											
Logistic	0.898(0.886-0.909)	0.831	0.840	0.835	0.826	0.837	0.661	0.661	0.840	0.820	0.611
Decision Tree	0.917(0.907-0.927)	0.841	0.863	0.827	0.856	0.845	0.682	0.684	0.863	0.819	0.683
Random Forest	1.000(1.000-1.000)	1.000	1.000	1.000	1.000	1.000	1.000	1.000	1.000	1.000	1.000
XGBoost	0.942(0.933-0.950)	0.872	0.882	0.872	0.873	0.877	0.744	0.744	0.882	0.861	0.744
LightGBM	0.997(0.996-0.998)	0.969	0.971	0.970	0.968	0.970	0.938	0.938	0.971	0.967	0.938
SVM	0.898(0.885-0.909)	0.832	0.840	0.838	0.825	0.839	0.663	0.663	0.840	0.823	0.663
ANN	0.900(0.888-0.912)	0.828	0.837	0.833	0.823	0.835	0.656	0.656	0.837	0.819	0.656
Validation set											
Logistic	0.893(0.873-0.912)	0.822	0.838	0.816	0.828	0.827	0.643	0.644	0.838	0.805	0.643
Decision Tree	0.879(0.857-0.898)	0.807	0.840	0.780	0.837	0.809	0.615	0.617	0.840	0.777	0.615
Random Forest	0.908(0.890-0.925)	0.831	0.840	0.835	0.826	0.838	0.661	0.661	0.840	0.821	0.661
XGBoost	0.911(0.893-0.927)	0.838	0.853	0.834	0.843	0.843	0.676	0.676	0.853	0.823	0.676
LightGBM	0.903(0.885-0.920)	0.827	0.843	0.821	0.833	0.832	0.654	0.655	0.843	0.810	0.654
SVM	0.893(0.873-0.912)	0.816	0.833	0.811	0.822	0.822	0.632	0.633	0.833	0.799	0.632
ANN	0.895(0.875-0.913)	0.819	0.840	0.808	0.831	0.823	0.638	0.639	0.840	0.798	0.638

AUROC, Area Under the Receiver Operating Characteristic Curve; CI, Confidence Interval; PPV, Positive Predictive Value; NPV, Negative Predictive Value; MCC, Matthews correlation coefficient; Logistic, Logistic Regression; Decision Tree, Decision Tree; Random Forest, Random Forest; XGBoost, Extreme Gradient Boosting; LightGBM, Light Gradient Boosting Machine; SVM, Support Vector Machine; ANN, Artificial Neural Network.

All classification metrics (accuracy, sensitivity, specificity, PPV, NPV, F1 score, Kappa, Youden’s J, MCC) were calculated using the default threshold of 0.5.

**Figure 6:**
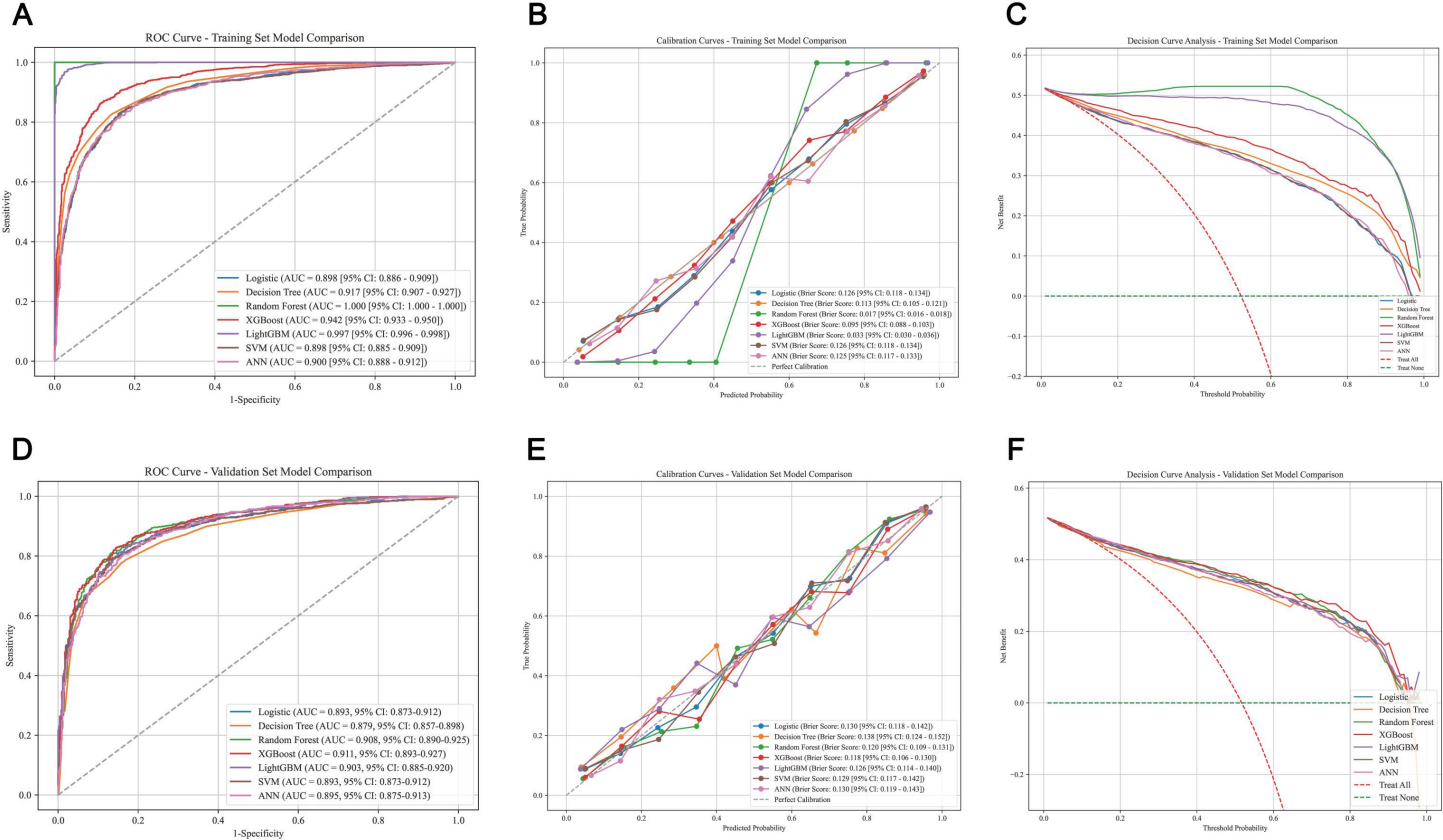
Assessment of model performance on the training set and validation set. **(A)** Receiver Operating Characteristic (ROC) curves on the Training Set. **(B)**Calibration curves on the Training Set. **(C)** Decision Curve Analysis (DCA) on the Training Set. **(D)** Receiver Operating Characteristic (ROC) curves on the Validation Cohort. **(E)** Calibration curves on the Validation Cohort. **(F)** Decision Curve Analysis (DCA) on the Validation Cohort. AUC, area under the curve; CI, confidence interval; XGBoost, eXtreme Gradient Boosting; LightGBM, Light Gradient Boosting Machine; SVM, support vector machine; ANN, artificial neural network.

As shown in **Figure 6**, the ROC curves for the training and validation sets (**Figures 6A, D**) further confirmed the excellent discriminative ability of XGBoost. The calibration performance of the XGBoost model was satisfactory in both training and validation cohorts, as reflected by Brier scores of 0.095 and 0.118, respectively (**Figures 6B, E**). According to decision curve analysis (**Figures 6C, F**), the XGBoost model yielded a superior net benefit compared with alternative strategies across a wide range of clinically relevant threshold probabilities.

Considering model stability, discriminative performance, and clinical interpretability, the XGBoost model was finally selected as the optimal model for predicting CVD risk. Hyperparameter tuning and model stability assessment were performed exclusively on the training set using five−fold stratified cross−validation. For each candidate set of hyperparameters, the training set was split into five subsets; four folds were used for training and the remaining fold for validation in a round−robin manner. The average AUC across the five validation folds was calculated, and the hyperparameter combination yielding the highest average AUC was selected. A grid search strategy (GridSearchCV) was employed. The final model was retrained on the entire training set using the optimal hyperparameters and then evaluated on the independent internal validation set. Details on hyperparameter optimization are provided in **Supplementary Table 7**.

To further evaluate the statistical significance of differences in AUC between models, the DeLong test was used for pairwise comparisons of all models in both the training and validation sets (**Supplementary Table 8**). In the validation set, XGBoost achieved an AUC significantly higher than logistic regression (P = 0.0005), decision tree (P < 0.001), LightGBM (P < 0.001), SVM (P = 0.0006), and ANN (P = 0.0081); however, the difference compared to random forest was not statistically significant (P = 0.1323). Although the absolute difference in AUC between XGBoost and logistic regression was only 0.018, the DeLong test confirmed that this difference was statistically significant, indicating that XGBoost indeed outperformed the simpler logistic regression model in discriminative performance.

### 3.9 Model interpretability analysis and web deployment

**Figure 7:**
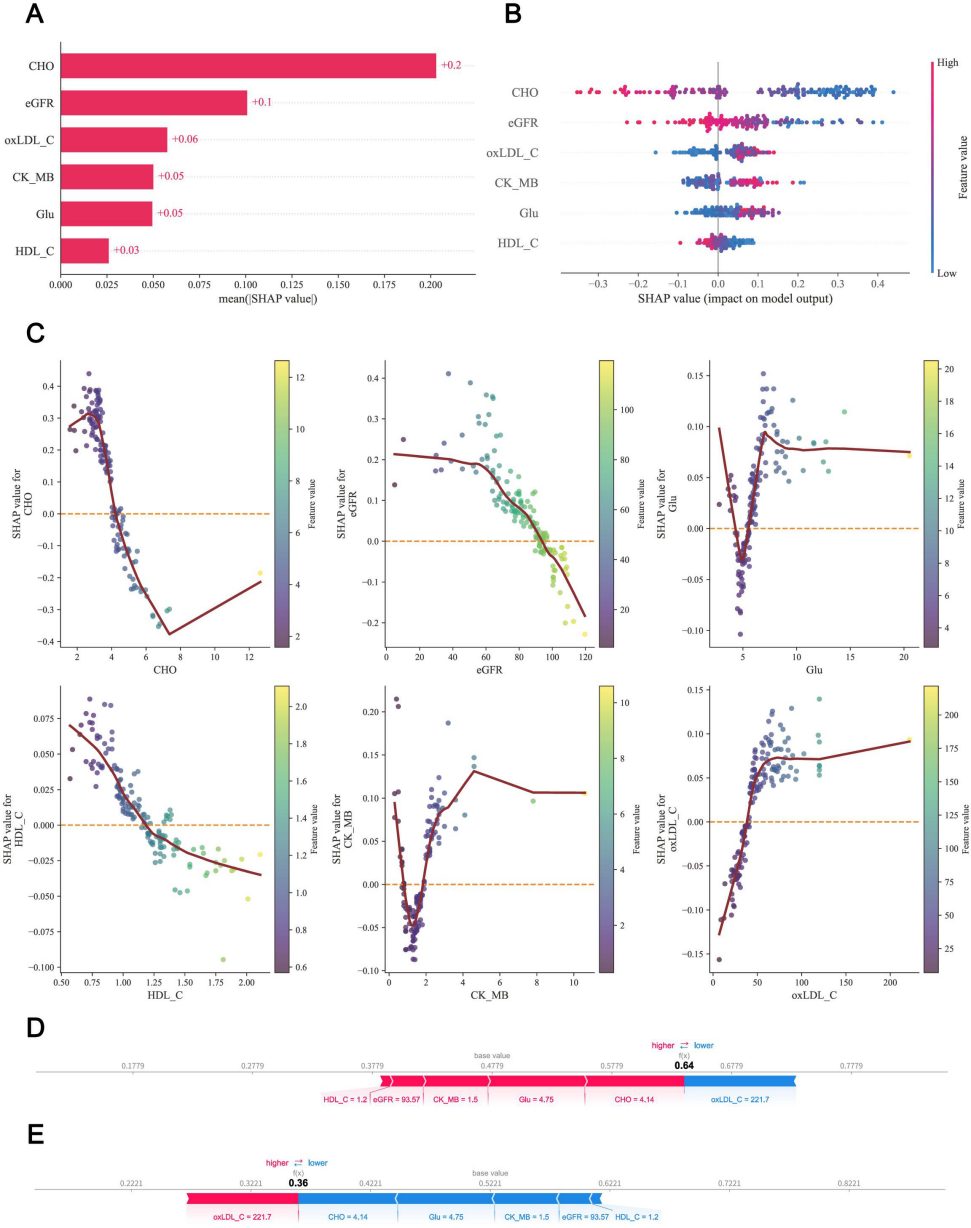
Interpretability analysis of the best-performing model (XGBoost, Extreme Gradient Boosting). **(A)** Global feature importance bar plot based on SHapley Additive exPlanations (SHAP) values. **(B)** Feature importance summary plot (beeswarm plot). **(C)** Feature dependence plots. **(D, E)** Individual prediction explanation force plots. SHAP, Shapley Additive exPlanations; CHO, total cholesterol; eGFR, estimated glomerular filtration rate; oxLDL-C, oxidized low-density lipoprotein cholesterol; CK-MB, creatine kinase-MB; Glu, glucose; HDL-C, high-density lipoprotein cholesterol.

To open the “black box” of the optimal XGBoost model, we applied the SHAP (SHapley Additive exPlanations) method for interpretability (**Figure 7**). As illustrated in **Figure 7A**, total cholesterol (CHO) ranked highest in global feature importance (mean |SHAP| value = 0.20), followed by eGFR, oxLDL-C, CK-MB, fasting glucose (Glu), and HDL-C. The beeswarm-style summary plot (**Figure 7B**) shows how each variable influences the prediction: red-colored higher values of Glu, oxLDL-C, and CK-MB are associated with elevated risk (positive SHAP), whereas higher HDL-C values mostly exert protective effects (negative SHAP). The dependence plots (**Figure 7C**) further depict the non-linear, dose-response patterns between key continuous features (e.g., oxLDL-C) and the model’s output. Finally, force plots for representative individuals (**Figure 7D**: high-risk case, predicted probability = 0.64; **Figure 7E**: low-risk case, probability = 0.36) break down the contribution of each feature to that patient’s risk estimate.

In order to bridge the gap between model development and clinical practice, we have developed a free access percentage based online risk assessment tool based on the XGBoost model. Clinical doctors can access https://machine11.shinyapps.io/oxldl/Used for fast and personalized CVD probability estimation.

## 4 Discussion

This study confirms that oxidized low-density lipoprotein cholesterol (oxLDL-C) is an effective biomarker for aiding the diagnosis of cardiovascular disease (CVD), demonstrating superior discriminatory performance compared to traditional markers like low-density lipoprotein cholesterol (LDL-C), triglycerides (TG), and the TG/HDL-C ratio. By integrating oxLDL-C with five other routine clinical indicators and applying the XGBoost machine learning algorithm, the constructed diagnostic model exhibited excellent discriminative performance (AUC = 0.911) and good calibration in internal validation. This research can assist in achieving more precise CVD diagnosis and patient screening in clinical practice.

Compared to traditional lipid markers, oxLDL-C provides more specific risk information from a mechanistic perspective. Traditional lipid management centers on LDL-C, yet it fails to fully capture a key pro-atherogenic step lipoproteins undergo *in vivo*—oxidative modification (24, 25). Previous studies have indicated that oxLDL-C is associated with cardiovascular disease independently of other lipid markers (26–28) and offers a significant advantage in distinguishing CVD patients from non-patients (29, 30), consistent with our findings. OxLDL is the active form of LDL resulting from oxidative stress; it is taken up by macrophages via scavenger receptor pathways, directly driving foam cell formation (31, 32). Simultaneously, oxLDL itself is a potent inflammatory activator, inducing adhesion molecule expression on endothelial cells and promoting cytokine release, thereby accelerating the initiation and progression of atherosclerotic plaques (33). The significant positive association and the distinct non-linear dose-response relationship between oxLDL-C and CVD risk observed in this study corroborate its central pathogenic role from a clinical epidemiological perspective. Incremental value analysis further showed that, beyond a traditional clinical model incorporating age, sex, BMI, and chronic disease history, oxLDL−C still provided statistically significant incremental predictive information (validation set: NRI = 0.469, IDI = 0.032, both P < 0.001), indicating that oxLDL−C carries independent discriminative information not captured by traditional variables. This provides quantitative justification for its inclusion in a CVD−aided diagnostic model.

In terms of model construction, the XGBoost algorithm employed in this study demonstrated high predictive performance through ensemble learning and regularization techniques. Its stability was quantitatively supported by the five−fold cross−validation results, indicating that the model performance is insensitive to different partitions of the training data and thus possesses good stability. Its overall performance surpassed that of comparison models like Logistic Regression and Support Vector Machine, reflecting good generalizability. The value of this model stems not only from the core indicator oxLDL-C but also from its successful integration of routine indicators reflecting multi-system physiological states. SHAP interpretability analysis revealed that total cholesterol (CHO), estimated glomerular filtration rate (eGFR), and oxLDL-C were the three most contributive features. Other selected indicators, such as elevated fasting glucose (Glu) (reflecting abnormal glucose metabolism and accumulation of advanced glycation end-products (34, 35)), elevated creatine kinase-MB (CK-MB) (indicating myocardial cell stress or injury (36)), and decreased high-density lipoprotein cholesterol (HDL-C) (impairing reverse cholesterol transport and anti-inflammatory functions (37–39)), each carry clear pathophysiological significance. By integrating information on lipid metabolism, oxidative stress, glucose metabolism, renal function (40), and myocardial injury, the model’s decision logic aligns with the complex, multi-factorial nature of CVD, thereby enhancing its biological plausibility and clinical credibility.

A major strength of this model lies in its practical clinical utility and potential for resource optimization. All included indicators are routinely measured in clinical laboratories across various healthcare levels, eliminating the need for expensive or specialized tests. This makes the model particularly suitable for resource-limited regions, primary care settings, or primary healthcare scenarios in less developed countries. To address diagnostic accessibility, we deployed the trained XGBoost model as a publicly available, free online tool. Clinicians can simply input a few readily available routine results to rapidly obtain an individualized CVD risk prediction. This model can serve as an exploratory diagnostic aid, offering clinicians preliminary risk−reference information; however, its clinical translation should proceed with caution. If further validated by multicenter prospective studies, this tool may in the future play a role in smart triage in primary care settings—reducing unnecessary follow−up tests for low−risk individuals while prompting timely referral for high−risk individuals. Nevertheless, until sufficient external validation, prospective validation, and health economic evaluation evidence are obtained, it should not be used as a direct basis for clinical decision−making. Subgroup analysis further indicated that the association between oxLDL-C and CVD risk remained robust across different ages, sexes, BMIs, and common chronic disease statuses, with no significant interactions detected. This suggests the general applicability of this biomarker and the constructed model across diverse populations.

This study has several limitations. First, the retrospective single−center design may introduce selection bias. Second, due to data source restrictions, we were unable to include information on patients’ lifestyle and medication history, which may have confounding effects on the effect estimates. Furthermore, the actual clinical utility of this model—i.e., whether it improves diagnostic efficiency or patient outcomes compared to current guideline−directed pathways—has not yet been evaluated in prospective implementation studies, and its real−world effectiveness requires further validation. Finally, regarding the methodology of feature selection, although this study alleviated the issue of feature redundancy through a preliminary correlation filtering step, the recursive feature elimination (RFE) method still exhibits dependence on the base model. Future model iterations could consider incorporating model−independent filter−based algorithms such as maximum relevance and minimum redundancy (mRMR), and systematically compare the impact of different feature selection strategies on model performance and stability, in order to further optimize the objectivity and interpretability of the variable combination.

## 5 Conclusion

In summary, this study confirms the value of oxLDL-C in CVD diagnosis and, by integrating it with routine indicators using machine learning, constructs an efficient, stable, and clinically accessible diagnostic tool. Achieving more accurate initial patient identification and triage at a relatively low overall cost can help optimize diagnostic pathways and improve resource allocation in resource-constrained healthcare environments.

## Data Availability

The raw data supporting the conclusions of this article will be made available by the authors, without undue reservation.

## Glossary

AG: Anion Gap
AG-Ratio: Albumin-to-Globulin Ratio
ALB: Albumin
ALP: Alkaline Phosphatase
ALT: Alanine Aminotransferase
ANN: Artificial Neural Network
AST: Aspartate Aminotransferase
AUC: Area Under the Curve
BAS: Basophil Count
BMI: Body Mass Index
ChE: Cholinesterase
CHG: Cholesterol-Glucose Index
CHO: Total Cholesterol
CI: Confidence Interval
CK: Creatine Kinase
CK-MB: Creatine Kinase-MB
CVD: Cardiovascular Disease
DCA: Decision Curve Analysis
DM: Diabetes Mellitus
dNLR: Derived Neutrophil-to-Lymphocyte Ratio
eGFR: Estimated Glomerular Filtration Rate
EOS: Eosinophil Count
GA: Glycated Albumin
GGT: Gamma-Glutamyl Transferase
Glu: Glucose
Hb: Hemoglobin
HCY: Homocysteine
HCT: Hematocrit
HDL-C: High-Density Lipoprotein Cholesterol
hs-CRP: High-Sensitivity C-Reactive Protein
LD: Lactate Dehydrogenase
LDL-C: Low-Density Lipoprotein Cholesterol
LightGBM: Light Gradient Boosting Machine
LYM: Lymphocyte Count
MCH: Mean Corpuscular Hemoglobin
MCHC: Mean Corpuscular Hemoglobin Concentration
MCV: Mean Corpuscular Volume
MLR: Monocyte-to-Lymphocyte Ratio
MONO: Monocyte Count
MPV: Mean Platelet Volume
NEUT: Neutrophil Count
NLR: Neutrophil-to-Lymphocyte Ratio
NMLR: Neutrophil-Monocyte-to-Lymphocyte Ratio
NPV: Negative Predictive Value
NT-proBNP: N-Terminal Pro-Brain Natriuretic Peptide
OR: Odds Ratio
oxLDL-C: Oxidized Low-Density Lipoprotein Cholesterol
PA: Prealbumin
PCT: Procalcitonin
PDW: Platelet Distribution Width
PHR: Platelet-to-Hemoglobin Ratio
P-LCR: Platelet-Large Cell Ratio
PLR: Platelet-to-Lymphocyte Ratio
PLT: Platelet Count
PPV: Positive Predictive Value
RBC: Red Blood Cell Count
RCS: Restricted Cubic Spline
RDW-CV: Red Cell Distribution Width-Coefficient of Variation
RDW-SD: Red Cell Distribution Width-Standard Deviation
RFE: Recursive Feature Elimination
ROC: Receiver Operating Characteristic
SD: Standard Deviation
SEN: Sensitivity
SHAP: SHapley Additive exPlanations
SII: Systemic Immune-Inflammation Index
SIRI: Systemic Inflammation Response Index
SPE: Specificity
SVM: Support Vector Machine
TB: Total Bilirubin
TBA: Total Bile Acids
TG: Triglycerides
TG/HDL-C: Triglyceride to High-Density Lipoprotein Cholesterol Ratio
TyG: Triglyceride-Glucose Index
UA: Uric Acid
WBC: White Blood Cell Count
XGBoost: Extreme Gradient Boosting.
