# Supplementary material for: Optimizing cardiovascular disease diagnosis through machine learning models integrating oxidized low-density lipoprotein and routine clinical indicators
Source: Front Endocrinol (Lausanne). 2026 Jul 22;17:1850695. doi: 10.3389/fendo.2026.1850695 (PMC13437373; doi:10.3389/fendo.2026.1850695)
Supplement: Supplementary file 1 [file SupplementaryFile1.docx]

**Supplementary materials (merged version)**

**Supplementary Table**

| **Supplementary Table S1. Missing Ratio of Variables in the Study** | |
| --- | --- |
| Variable | Missing Ratio |
| Outcome | 0 |
| Sex | 0 |
| DM | 0 |
| Hyperlipidemia | 0 |
| Age | 0 |
| BMI | 0.0824742 |
| oxLDL-C | 0 |
| GGT | 0 |
| ALB | 0 |
| ALT | 0 |
| hs-CRP | 0 |
| ChE | 0 |
| LDL-C | 0.0358111 |
| TG | 0 |
| HDL-C | 0 |
| CK | 0 |
| CK-MB | 0.0512333 |
| ALP | 0 |
| UA | 0 |
| Glu | 0 |
| PA | 0 |
| LD | 0 |
| GA | 0 |
| AST | 0 |
| Hcy | 0 |
| DB | 0 |
| CHO | 0 |
| TB | 0.0496473 |
| eGFR | 0 |
| AG-Ratio | 0 |
| AG | 0 |
| TBA | 0 |
| WBC | 0 |
| RBC | 0 |
| Hb | 0 |
| HCT | 0 |
| MCV | 0 |
| MCH | 0 |
| MCHC | 0 |
| PLT | 0 |
| MPV | 0 |
| NEU | 0 |
| LYM | 0 |
| MON | 0 |
| EOS | 0 |
| BAS | 0 |
| PCT | 0 |
| PDW | 0 |
| RDW-SD | 0 |
| RDW-CV | 0 |
| P-LCR | 0 |
| NT-proBNP | 0.0626695 |
| NLR | 0 |
| PLR | 0 |
| TyG | 0 |
| CHG | 0 |
| PHR | 0 |
| dNLR | 0 |
| MLR | 0 |
| NMLR | 0 |
| SIRI | 0 |
| SII | 0 |

| **Supplementary Table S2. Baseline Characteristics of the Training Set** | | | | |  |
| --- | --- | --- | --- | --- | --- |
|  |  |  |  |  |  |
| Variables | Total(n=2581) | Without CVD(n=1233) | With CVD(n=1348) | *p* |  |
| Age | 57.00 [46.00, 66.00] | 52.00 [42.00, 62.00] | 61.00 [52.00, 68.00] | <0.001 |  |
| BMI | 24.90 [22.80, 27.00] | 25.10 [23.10, 27.30] | 24.70 [22.58, 26.80] | <0.001 |  |
| Sex | 1028 (39.83) | 600 ( 48.66) | 428 (31.75) | <0.001 |  |
| Male | 1553 (60.17) | 633 ( 51.34) | 920 (68.25) |  |  |
| Female | 1028 (39.83) | 600 ( 48.66) | 428 (31.75) |  |  |
| DM | 1459 (56.53) | 529 ( 42.90) | 930 (68.99) | <0.001 |  |
| Hyperlipidemia | 693 (26.85) | 330 ( 26.76) | 363 (26.93) | 0.96 |  |
| stroke | 396 (15.34) | 0 ( 0.00) | 396 (29.38) | <0.001 |  |
| heart disease | 1326 (51.38) | 0 ( 0.00) | 1326 (98.37) | <0.001 |  |
| oxLDL-C(U/L) | 39.37 [28.15, 52.08] | 34.52 [24.70, 46.30] | 43.10 [32.38, 56.18] | <0.001 |  |
| GGT(U/L) | 23.00 [15.00, 36.00] | 21.00 [13.00, 33.00] | 24.15 [17.00, 38.00] | <0.001 |  |
| ALB(g/dl) | 4.54 [4.31, 4.75] | 4.61 [4.43, 4.80] | 4.48 [4.19, 4.71] | <0.001 |  |
| ALT(U/L) | 19.00 [13.00, 28.60] | 18.00 [12.00, 27.00] | 20.00 [14.00, 30.02] | <0.001 |  |
| hs-CRP(mg/L) | 0.84 [0.47, 1.83] | 0.76 [0.45, 1.54] | 0.92 [0.50, 2.20] | <0.001 |  |
| ChE(kU/L) | 9.00 [7.80, 10.10] | 9.20 [8.00, 10.30] | 8.80 [7.60, 10.00] | <0.001 |  |
| LDL-C(mmol/L) | 3.06 [2.48, 3.62] | 3.03 [2.45, 3.59] | 3.08 [2.49, 3.66] | 0.108 |  |
| TG(mmol/L) | 1.29 [0.94, 1.83] | 1.27 [0.91, 1.87] | 1.30 [0.96, 1.79] | 0.324 |  |
| HDL-C(mmol/L) | 1.21 [1.01, 1.48] | 1.34 [1.12, 1.60] | 1.12 [0.94, 1.34] | <0.001 |  |
| CK(U/L) | 84.00 [62.00, 116.00] | 86.00 [65.00, 117.00] | 82.00 [59.00, 114.00] | 0.004 |  |
| CK-MB(ng/ml) | 1.50 [1.10, 2.00] | 1.40 [1.10, 1.70] | 1.70 [1.20, 2.30] | <0.001 |  |
| ALP(U/L) | 72.00 [60.00, 86.00] | 68.00 [57.00, 82.00] | 76.00 [64.00, 91.00] | <0.001 |  |
| UA(umol/L) | 320.90 [263.70, 381.80] | 320.10 [263.50, 384.60] | 322.45 [263.78, 380.63] | 0.687 |  |
| Glu(mmol/L) | 5.38 [4.95, 6.13] | 5.19 [4.86, 5.63] | 5.67 [5.12, 6.61] | <0.001 |  |
| PA (g/L) | 0.26 [0.23, 0.30] | 0.27 [0.24, 0.30] | 0.26 [0.22, 0.30] | <0.001 |  |
| LD (U/L) | 171.00 [153.00, 191.20] | 166.00 [150.00, 184.00] | 177.00 [157.98, 200.00] | <0.001 |  |
| GA (g/dl) | 0.54 [0.49, 0.61] | 0.53 [0.49, 0.58] | 0.55 [0.49, 0.64] | <0.001 |  |
| AST(U/L) | 19.00 [16.00, 24.00] | 18.00 [15.00, 22.00] | 20.75 [17.00, 26.00] | <0.001 |  |
| HCY(umol/L) | 11.40 [9.30, 14.20] | 11.00 [9.10, 13.70] | 11.80 [9.50, 14.70] | <0.001 |  |
| DB(umol/L) | 4.09 [3.17, 5.35] | 3.85 [3.04, 4.89] | 4.40 [3.30, 5.90] | <0.001 |  |
| CHO(mmol/L) | 4.36 [3.47, 5.24] | 5.09 [4.43, 5.71] | 3.57 [3.05, 4.36] | <0.001 |  |
| TB(umol/L) | 11.33 [8.56, 15.18] | 10.36 [7.86, 13.78] | 12.37 [9.40, 16.26] | <0.001 |  |
| eGFR(mL/min/1.73m^2) | 93.50 [80.36, 103.50] | 100.46 [90.90, 109.08] | 86.68 [72.99, 96.91] | <0.001 |  |
| AG-Ratio | 1.65 [1.50, 1.83] | 1.62 [1.48, 1.78] | 1.70 [1.52, 1.87] | <0.001 |  |
| AG(mmol/L) | 16.40 [14.30, 18.00] | 16.50 [14.80, 18.20] | 16.20 [13.60, 17.80] | <0.001 |  |
| TBA(umol/L) | 3.10 [2.10, 4.80] | 3.00 [2.00, 4.60] | 3.20 [2.10, 4.90] | 0.051 |  |
| WBC(×10^9/L) | 6.55 [5.50, 7.83] | 6.34 [5.35, 7.55] | 6.73 [5.70, 8.13] | <0.001 |  |
| RBC(×10^12/L) | 4.80 [4.42, 5.16] | 4.85 [4.50, 5.19] | 4.74 [4.36, 5.12] | <0.001 |  |
| Hb(g/L) | 147.00 [134.00, 157.00] | 148.00 [135.00, 158.00] | 146.00 [133.00, 156.00] | <0.001 |  |
| HCT | 43.20 [39.90, 46.20] | 43.60 [40.30, 46.30] | 43.00 [39.60, 46.00] | 0.001 |  |
| MCV(fL) | 90.00 [87.50, 92.70] | 89.70 [87.10, 92.10] | 90.30 [88.00, 93.10] | <0.001 |  |
| MCH(pg) | 30.50 [29.50, 31.50] | 30.40 [29.40, 31.30] | 30.60 [29.60, 31.60] | <0.001 |  |
| MCHC(g/L) | 338.00 [333.00, 343.00] | 339.00 [333.00, 344.00] | 338.00 [333.00, 343.00] | 0.015 |  |
| PLT(×10^9/L) | 219.00 [182.00, 260.00] | 231.00 [198.00, 273.00] | 207.00 [172.00, 246.00] | <0.001 |  |
| MPV(fL) | 9.50 [8.80, 10.30] | 9.50 [8.90, 10.20] | 9.50 [8.80, 10.30] | 0.607 |  |
| NEUT(×10^9/L) | 3.99 [3.17, 5.10] | 3.80 [2.99, 4.73] | 4.24 [3.35, 5.37] | <0.001 |  |
| LYM(×10^9/L) | 1.84 [1.45, 2.25] | 1.89 [1.52, 2.33] | 1.79 [1.39, 2.19] | <0.001 |  |
| MONO(×10^9/L) | 0.38 [0.30, 0.47] | 0.36 [0.29, 0.44] | 0.39 [0.31, 0.49] | <0.001 |  |
| EOS(×10^9/L) | 0.12 [0.07, 0.20] | 0.12 [0.07, 0.20] | 0.11 [0.06, 0.20] | 0.091 |  |
| BAS(×10^9/L) | 0.02 [0.02, 0.04] | 0.02 [0.02, 0.04] | 0.02 [0.02, 0.03] | 0.004 |  |
| PCT | 0.21 [0.18, 0.25] | 0.22 [0.19, 0.26] | 0.20 [0.17, 0.23] | <0.001 |  |
| PDW | 16.10 [15.90, 16.40] | 16.10 [15.90, 16.40] | 16.20 [15.90, 16.40] | 0.458 |  |
| RDW-SD(fl) | 41.90 [40.40, 43.60] | 41.50 [40.10, 43.00] | 42.30 [40.70, 44.02] | <0.001 |  |
| RDW-CV | 13.00 [12.60, 13.40] | 12.90 [12.50, 13.30] | 13.00 [12.60, 13.50] | <0.001 |  |
| P-LCR | 22.80 [18.20, 28.10] | 22.60 [18.40, 27.80] | 23.00 [18.10, 28.40] | 0.544 |  |
| NT-proBNP(pg/mL) | 103.00 [39.00, 458.00] | 74.00 [30.00, 223.00] | 157.21 [51.38, 815.59] | <0.001 |  |
| NLR | 2.14 [1.64, 2.94] | 2.01 [1.54, 2.66] | 2.31 [1.77, 3.27] | <0.001 |  |
| PLR | 120.80 [94.04, 154.84] | 123.12 [96.86, 154.63] | 118.10 [91.00, 155.16] | 0.031 |  |
| TyG | 8.66 [8.31, 9.07] | 8.58 [8.21, 9.04] | 8.72 [8.39, 9.09] | <0.001 |  |
| CHG | 5.15 [4.91, 5.41] | 5.17 [4.93, 5.44] | 5.13 [4.88, 5.39] | 0.004 |  |
| PHR | 176.77 [138.85, 228.81] | 171.97 [134.85, 219.74] | 182.13 [143.46, 236.54] | <0.001 |  |
| dNLR | 0.88 [0.85, 0.90] | 0.88 [0.85, 0.90] | 0.88 [0.86, 0.91] | 0.001 |  |
| MLR | 0.20 [0.16, 0.27] | 0.19 [0.15, 0.24] | 0.22 [0.17, 0.29] | <0.001 |  |
| NMLR | 2.34 [1.82, 3.20] | 2.20 [1.71, 2.90] | 2.53 [1.96, 3.54] | <0.001 |  |
| SIRI | 0.81 [0.55, 1.24] | 0.73 [0.50, 1.05] | 0.91 [0.61, 1.44] | <0.001 |  |
| SII | 470.75 [339.58, 690.08] | 462.05 [336.84, 657.00] | 484.40 [344.83, 716.91] | 0.002 |  |

| **Supplementary Table S3. Baseline Characteristics of the Validation Set** | | | | |  |
| --- | --- | --- | --- | --- | --- |
|  |  |  |  |  |  |
| Variables | Total(n=1105) | Without CVD(n=528) | With CVD(n=577) | *p* |  |
| Age | 57.00 [46.00, 67.00] | 53.00 [42.00, 63.00] | 61.00 [52.00, 68.00] | <0.001 |  |
| BMI | 24.70 [22.50, 26.70] | 25.10 [23.00, 27.00] | 24.40 [22.10, 26.50] | 0.001 |  |
| Sex | 470 (42.53) | 259 ( 49.05) | 211 (36.57) | <0.001 |  |
| Male | 635 (57.47) | 269 ( 50.95) | 366 (63.43) |  |  |
| Female | 470 (42.53) | 259 ( 49.05) | 211 (36.57) |  |  |
| DM | 605 (54.75) | 215 ( 40.72) | 390 (67.59) | <0.001 |  |
| Hyperlipidemia | 270 (24.43) | 130 ( 24.62) | 140 (24.26) | 0.946 |  |
| stroke | 167 (15.11) | 0 ( 0.00) | 167 (28.94) | <0.001 |  |
| heart disease | 569 (51.49) | 0 ( 0.00) | 569 (98.61) | <0.001 |  |
| oxLDL-C(U/L) | 38.88 [27.40, 51.73] | 34.18 [24.25, 45.00] | 44.10 [32.51, 56.85] | <0.001 |  |
| GGT(U/L) | 23.00 [15.00, 36.60] | 20.00 [14.00, 32.25] | 26.00 [17.00, 41.00] | <0.001 |  |
| ALB(g/dl) | 4.54 [4.29, 4.75] | 4.64 [4.46, 4.80] | 4.42 [4.14, 4.67] | <0.001 |  |
| ALT(U/L) | 19.00 [13.00, 29.00] | 18.00 [13.00, 26.00] | 21.00 [14.00, 31.00] | 0.001 |  |
| hs-CRP(mg/L) | 0.92 [0.49, 2.20] | 0.81 [0.47, 1.78] | 1.03 [0.52, 2.70] | <0.001 |  |
| ChE(kU/L) | 8.90 [7.70, 10.00] | 9.20 [8.20, 10.30] | 8.50 [7.50, 9.70] | <0.001 |  |
| LDL-C(mmol/L) | 3.09 [2.50, 3.65] | 3.04 [2.54, 3.57] | 3.14 [2.45, 3.70] | 0.531 |  |
| TG(mmol/L) | 1.33 [0.96, 1.84] | 1.33 [0.96, 1.87] | 1.33 [0.95, 1.82] | 0.628 |  |
| HDL-C(mmol/L) | 1.23 [1.04, 1.46] | 1.35 [1.14, 1.60] | 1.14 [0.97, 1.34] | <0.001 |  |
| CK(U/L) | 83.00 [62.00, 118.00] | 85.50 [65.75, 120.00] | 81.80 [59.70, 113.00] | 0.016 |  |
| CK-MB(ng/ml) | 1.50 [1.10, 2.10] | 1.40 [1.10, 1.90] | 1.70 [1.20, 2.30] | <0.001 |  |
| ALP(U/L) | 74.00 [61.00, 88.00] | 71.00 [58.00, 85.00] | 76.00 [64.00, 92.00] | <0.001 |  |
| UA(umol/L) | 317.70 [262.00, 376.90] | 318.65 [261.48, 379.92] | 317.20 [262.50, 375.10] | 0.609 |  |
| Glu(mmol/L) | 5.39 [4.95, 6.09] | 5.18 [4.86, 5.68] | 5.67 [5.05, 6.64] | <0.001 |  |
| PA (g/L) | 0.26 [0.23, 0.30] | 0.27 [0.24, 0.30] | 0.25 [0.22, 0.29] | <0.001 |  |
| LD (U/L) | 173.60 [155.00, 195.00] | 168.00 [153.00, 186.25] | 179.00 [158.00, 204.00] | <0.001 |  |
| GA (g/dl) | 0.54 [0.49, 0.60] | 0.53 [0.49, 0.58] | 0.56 [0.49, 0.63] | <0.001 |  |
| AST(U/L) | 19.40 [16.00, 24.00] | 19.00 [16.00, 23.00] | 20.00 [17.00, 25.00] | 0.001 |  |
| HCY(umol/L) | 11.60 [9.30, 14.70] | 11.00 [9.10, 13.10] | 12.40 [9.70, 15.90] | <0.001 |  |
| DB(umol/L) | 4.05 [3.10, 5.32] | 3.72 [2.94, 4.72] | 4.40 [3.38, 5.87] | <0.001 |  |
| CHO(mmol/L) | 4.43 [3.50, 5.26] | 5.08 [4.47, 5.71] | 3.59 [3.11, 4.41] | <0.001 |  |
| TB(umol/L) | 11.10 [8.15, 14.97] | 10.03 [7.58, 13.59] | 11.98 [9.15, 16.45] | <0.001 |  |
| eGFR(mL/min/1.73m^2) | 93.11 [80.44, 103.15] | 99.75 [89.62, 108.23] | 86.98 [72.80, 96.34] | <0.001 |  |
| AG-Ratio | 1.66 [1.50, 1.83] | 1.62 [1.48, 1.77] | 1.71 [1.55, 1.91] | <0.001 |  |
| AG(mmol/L) | 16.20 [14.30, 17.90] | 16.50 [14.97, 18.10] | 15.90 [13.50, 17.70] | <0.001 |  |
| TBA(umol/L) | 3.10 [2.00, 4.80] | 3.00 [2.00, 4.50] | 3.20 [2.10, 5.20] | 0.02 |  |
| WBC(×10^9/L) | 6.51 [5.42, 7.74] | 6.42 [5.45, 7.67] | 6.57 [5.41, 7.89] | 0.102 |  |
| RBC(×10^12/L) | 4.83 [4.44, 5.16] | 4.90 [4.52, 5.26] | 4.73 [4.32, 5.10] | <0.001 |  |
| Hb(g/L) | 147.00 [134.00, 158.00] | 149.00 [137.00, 159.00] | 145.00 [130.00, 157.00] | <0.001 |  |
| HCT | 43.30 [39.80, 46.60] | 44.00 [40.77, 47.00] | 42.80 [39.10, 46.10] | <0.001 |  |
| MCV(fL) | 89.80 [87.70, 92.30] | 89.60 [87.40, 91.70] | 90.10 [88.10, 92.80] | 0.001 |  |
| MCH(pg) | 30.50 [29.50, 31.30] | 30.40 [29.40, 31.20] | 30.50 [29.60, 31.50] | 0.029 |  |
| MCHC(g/L) | 338.00 [333.00, 343.00] | 339.00 [333.00, 343.00] | 338.00 [332.00, 342.00] | 0.062 |  |
| PLT(×10^9/L) | 220.00 [185.00, 259.00] | 235.00 [198.75, 269.00] | 211.00 [176.00, 247.00] | <0.001 |  |
| MPV(fL) | 9.50 [8.90, 10.20] | 9.50 [8.97, 10.20] | 9.60 [8.90, 10.20] | 0.695 |  |
| NEUT(×10^9/L) | 3.98 [3.22, 5.10] | 3.89 [3.16, 4.86] | 4.08 [3.25, 5.32] | 0.007 |  |
| LYM(×10^9/L) | 1.80 [1.40, 2.27] | 1.87 [1.51, 2.31] | 1.72 [1.28, 2.20] | <0.001 |  |
| MONO(×10^9/L) | 0.39 [0.31, 0.47] | 0.37 [0.30, 0.45] | 0.40 [0.32, 0.49] | 0.001 |  |
| EOS(×10^9/L) | 0.11 [0.06, 0.18] | 0.11 [0.07, 0.18] | 0.11 [0.06, 0.18] | 0.267 |  |
| BAS(×10^9/L) | 0.02 [0.02, 0.04] | 0.03 [0.02, 0.04] | 0.02 [0.02, 0.03] | 0.025 |  |
| PCT | 0.21 [0.18, 0.25] | 0.22 [0.19, 0.26] | 0.20 [0.17, 0.23] | <0.001 |  |
| PDW | 16.20 [15.90, 16.40] | 16.15 [15.90, 16.40] | 16.20 [15.90, 16.40] | 0.58 |  |
| RDW-SD(fl) | 41.90 [40.40, 43.50] | 41.50 [40.18, 43.00] | 42.20 [40.60, 43.90] | <0.001 |  |
| RDW-CV | 12.90 [12.60, 13.40] | 12.90 [12.60, 13.30] | 13.00 [12.60, 13.50] | 0.003 |  |
| P-LCR | 22.90 [18.80, 27.70] | 22.60 [18.90, 27.90] | 23.20 [18.60, 27.60] | 0.811 |  |
| NT-proBNP(pg/mL) | 111.00 [43.00, 447.00] | 87.00 [34.75, 282.77] | 142.00 [55.00, 676.00] | <0.001 |  |
| NLR | 2.17 [1.64, 3.01] | 2.00 [1.59, 2.67] | 2.34 [1.70, 3.65] | <0.001 |  |
| PLR | 121.11 [95.19, 163.64] | 122.08 [99.10, 156.69] | 120.09 [92.78, 165.99] | 0.716 |  |
| TyG | 8.71 [8.32, 9.07] | 8.64 [8.27, 9.02] | 8.75 [8.41, 9.09] | 0.004 |  |
| CHG | 5.15 [4.91, 5.43] | 5.18 [4.95, 5.45] | 5.11 [4.88, 5.41] | 0.01 |  |
| PHR | 176.07 [140.65, 229.35] | 171.65 [136.33, 223.28] | 182.11 [143.14, 233.33] | 0.027 |  |
| dNLR | 0.88 [0.85, 0.91] | 0.88 [0.85, 0.91] | 0.88 [0.85, 0.91] | 0.583 |  |
| MLR | 0.21 [0.16, 0.27] | 0.19 [0.16, 0.24] | 0.23 [0.17, 0.32] | <0.001 |  |
| NMLR | 2.38 [1.81, 3.26] | 2.18 [1.74, 2.90] | 2.57 [1.89, 3.98] | <0.001 |  |
| SIRI | 0.81 [0.57, 1.24] | 0.75 [0.55, 1.04] | 0.90 [0.61, 1.50] | <0.001 |  |
| SII | 473.90 [344.77, 717.39] | 468.36 [348.99, 652.82] | 487.62 [340.71, 780.61] | 0.199 |  |

| **Supplementary Table S4. Multivariate logistic regression** | | | | | | | |  |
| --- | --- | --- | --- | --- | --- | --- | --- | --- |
|  |  |  |  |  |  |  |  |  |
| Variable | Beta | SE | Wald | OR | Lower-CI | Upper-CI | p-value |  |
| Age | 0.054 | 0.006 | 9.199 | 1.055 | 1.043 | 1.067 | 0 |  |
| BMI | -0.07 | 0.022 | -3.22 | 0.933 | 0.894 | 0.973 | 0.001 |  |
| Sex | 0.897 | 0.149 | 6.015 | 2.453 | 1.831 | 3.286 | 0 |  |
| DM | 1.426 | 0.151 | 9.444 | 4.164 | 3.097 | 5.598 | 0 |  |
| oxLDL-C | 0.018 | 0.003 | 5.975 | 1.018 | 1.012 | 1.024 | 0 |  |
| GGT | 0.005 | 0.003 | 1.949 | 1.005 | 1 | 1.011 | 0.051 |  |
| ALB | -1.157 | 0.238 | -4.86 | 0.314 | 0.197 | 0.501 | 0 |  |
| ALT | -0.009 | 0.004 | -1.956 | 0.991 | 0.983 | 1 | 0.05 |  |
| hs-CRP | 0.071 | 0.023 | 3.096 | 1.073 | 1.026 | 1.123 | 0.002 |  |
| ChE | -0.047 | 0.047 | -0.991 | 0.954 | 0.87 | 1.047 | 0.322 |  |
| HDL-C | -2.797 | 0.863 | -3.24 | 0.061 | 0.011 | 0.331 | 0.001 |  |
| CK | -0.001 | 0.001 | -1.759 | 0.999 | 0.998 | 1 | 0.079 |  |
| CK-MB | 0.166 | 0.083 | 2.004 | 1.181 | 1.004 | 1.39 | 0.045 |  |
| ALP | 0.009 | 0.003 | 2.864 | 1.01 | 1.003 | 1.016 | 0.004 |  |
| Glu | 0.492 | 0.184 | 2.676 | 1.636 | 1.141 | 2.346 | 0.007 |  |
| PA | -3.159 | 1.571 | -2.01 | 0.042 | 0.002 | 0.924 | 0.044 |  |
| LD | 0.012 | 0.003 | 4.796 | 1.012 | 1.007 | 1.017 | 0 |  |
| GA | 0.368 | 0.833 | 0.442 | 1.445 | 0.283 | 7.387 | 0.658 |  |
| AST | 0.041 | 0.01 | 4.218 | 1.042 | 1.022 | 1.062 | 0 |  |
| Hcy | 0.005 | 0.008 | 0.633 | 1.005 | 0.989 | 1.022 | 0.527 |  |
| DB | -0.007 | 0.042 | -0.154 | 0.994 | 0.915 | 1.079 | 0.877 |  |
| CHO | -0.801 | 0.274 | -2.922 | 0.449 | 0.262 | 0.768 | 0.003 |  |
| TB | 0.029 | 0.014 | 2.098 | 1.029 | 1.002 | 1.057 | 0.036 |  |
| eGFR | -0.053 | 0.005 | -11.597 | 0.948 | 0.94 | 0.957 | 0 |  |
| AG-Ratio | 1.933 | 0.303 | 6.375 | 6.908 | 3.813 | 12.514 | 0 |  |
| AG | -0.174 | 0.027 | -6.465 | 0.84 | 0.797 | 0.886 | 0 |  |
| WBC | -0.436 | 0.405 | -1.077 | 0.646 | 0.292 | 1.43 | 0.281 |  |
| RBC | 0.849 | 1.934 | 0.439 | 2.338 | 0.053 | 103.422 | 0.661 |  |
| Hb | -0.054 | 0.158 | -0.341 | 0.947 | 0.695 | 1.292 | 0.733 |  |
| HCT | 0.033 | 0.608 | 0.054 | 1.033 | 0.314 | 3.405 | 0.957 |  |
| MCV | 0.269 | 0.28 | 0.959 | 1.308 | 0.756 | 2.265 | 0.338 |  |
| MCH | -0.53 | 0.773 | -0.685 | 0.589 | 0.129 | 2.68 | 0.493 |  |
| MCHC | 0.048 | 0.081 | 0.591 | 1.049 | 0.896 | 1.228 | 0.555 |  |
| PLT | -0.001 | 0.004 | -0.292 | 0.999 | 0.991 | 1.007 | 0.771 |  |
| NEU | 0.764 | 0.457 | 1.671 | 2.147 | 0.876 | 5.259 | 0.095 |  |
| LYM | 0.687 | 0.455 | 1.511 | 1.989 | 0.815 | 4.851 | 0.131 |  |
| MON | -1.061 | 1.186 | -0.895 | 0.346 | 0.034 | 3.538 | 0.371 |  |
| BAS | 0.611 | 3.963 | 0.154 | 1.843 | 0.001 | 4353.868 | 0.877 |  |
| PCT | -7.864 | 2.355 | -3.34 | 0 | 0 | 0.039 | 0.001 |  |
| NT-proBNP | 0 | 0 | 1.417 | 1 | 1 | 1 | 0.157 |  |
| NLR | 0.028 | 0.178 | 0.158 | 1.028 | 0.726 | 1.458 | 0.875 |  |
| PLR | -0.001 | 0.004 | -0.387 | 0.999 | 0.991 | 1.006 | 0.699 |  |
| TyG | 0.644 | 0.183 | 3.513 | 1.904 | 1.329 | 2.728 | 0 |  |
| CHG | -2.504 | 1.211 | -2.068 | 0.082 | 0.008 | 0.878 | 0.039 |  |
| PHR | -0.003 | 0.004 | -0.72 | 0.997 | 0.99 | 1.005 | 0.471 |  |
| dNLR | -3.952 | 2.235 | -1.768 | 0.019 | 0 | 1.535 | 0.077 |  |
| MLR | 4.668 | 1.837 | 2.541 | 106.522 | 2.909 | 3900.757 | 0.011 |  |
| NMLR | -0.169 | 0.203 | -0.833 | 0.845 | 0.568 | 1.257 | 0.405 |  |
| SIRI | -0.262 | 0.1 | -2.634 | 0.769 | 0.633 | 0.935 | 0.008 |  |
| SII | 0.001 | 0.001 | 1.549 | 1.001 | 1 | 1.002 | 0.121 |  |

Abbreviations: Beta, Regression coefficient; SE, Standard Error; Wald, Wald statistic; OR, Odds Ratio; Lower-CI, 95% Confidence Interval Lower Limit; Upper-CI, 95% Confidence Interval Upper Limit; BMI, Body Mass Index; DM, Diabetes Mellitus; oxLDL-C, Oxidized Low-Density Lipoprotein Cholesterol; GGT, Gamma-Glutamyl Transferase; ALB, Albumin; ALT, Alanine Aminotransferase; hs-CRP, High-Sensitivity C-Reactive Protein; ChE, Cholinesterase; HDL-C, High-Density Lipoprotein Cholesterol; CK, Creatine Kinase; CK-MB, Creatine Kinase Myocardial Band; ALP, Alkaline Phosphatase; Glu, Glucose; PA, Prealbumin; LD, Lactate Dehydrogenase; GA, Glycated Albumin; AST, Aspartate Aminotransferase; Hcy, Homocysteine; DB, Direct Bilirubin; CHO, Total Cholesterol; TB, Total Bilirubin; eGFR, Estimated Glomerular Filtration Rate; AG-Ratio, Albumin-to-Globulin Ratio; AG, Anion Gap; WBC, White Blood Cell Count; RBC, Red Blood Cell Count; Hb, Hemoglobin; HCT, Hematocrit; MCV, Mean Corpuscular Volume; MCH, Mean Corpuscular Hemoglobin; MCHC, Mean Corpuscular Hemoglobin Concentration; PLT, Platelet Count; NEU, Neutrophil Count; LYM, Lymphocyte Count; MON, Monocyte Count; BAS, Basophil Count; PCT, Procalcitonin; NT-proBNP, N-terminal pro-Brain Natriuretic Peptide; NLR, Neutrophil-to-Lymphocyte Ratio; PLR, Platelet-to-Lymphocyte Ratio; TyG, Triglyceride-Glucose Index; CHG, Cumulative Hyperglycemia Index; PHR, Platelet-to-Hemoglobin Ratio; dNLR, Derived Neutrophil-to-Lymphocyte Ratio; MLR, Monocyte-to-Lymphocyte Ratio; NMLR, Neutrophil-Monocyte-to-Lymphocyte Ratio; SIRI, Systemic Inflammation Response Index; SII, Systemic Immune-Inflammation Index.

| **Supplementary Table S5. Variance Inflation Factor test** | |
| --- | --- |
| Variable | VIF |
| CK_MB | 1.011544825 |
| oxLDL_C | 1.00972598 |
| Glu | 1.044894138 |
| CHO | 1.18355834 |
| eGFR | 1.067971228 |
| HDL_C | 1.181519302 |

Abbreviations: CHO, total cholesterol; eGFR, estimated glomerular filtration rate; oxLDL-C, oxidized low-density lipoprotein cholesterol; CK-MB, creatine kinase-MB; Glu, glucose; HDL-C, high-density lipoprotein cholesterol.

**Supplementary Table 6. Stability assessment of RFE feature selection via nested cross-validation.**

| Fold | AUC | Inner-AUC | Best-Params | Features |
| --- | --- | --- | --- | --- |
| 1 | 0.906 | 0.927 | {'C': 0.1} | ['Age', 'oxLDL-C', 'ALB', 'LDL-C', 'HDL-C', 'CK-MB', 'Glu', 'CHO', 'eGFR', 'NT-proBNP'] |
| 2 | 0.940 | 0.922 | {'C': 0.1} | ['Age', 'oxLDL-C', 'ALB', 'LDL-C', 'HDL-C', 'CK-MB', 'Glu', 'CHO', 'eGFR', 'NT-proBNP'] |
| 3 | 0.935 | 0.922 | {'C': 0.1} | ['Age', 'oxLDL-C', 'ALB', 'LDL-C', 'HDL-C', 'CK-MB', 'Glu', 'CHO', 'eGFR', 'NT-proBNP'] |
| 4 | 0.921 | 0.925 | {'C': 0.01} | ['Age', 'oxLDL-C', 'ALB', 'LDL-C', 'HDL-C', 'CK-MB', 'Glu', 'CHO', 'eGFR', 'NT-proBNP'] |
| 5 | 0.894 | 0.928 | {'C': 0.1} | ['Age', 'oxLDL-C', 'ALB', 'LDL-C', 'HDL-C', 'CK-MB', 'Glu', 'CHO', 'eGFR', 'NT-proBNP'] |
| 6 | 0.950 | 0.921 | {'C': 10.0} | ['Age', 'oxLDL-C', 'ALB', 'LDL-C', 'HDL-C', 'CK-MB', 'Glu', 'CHO', 'eGFR', 'NT-proBNP'] |
| 7 | 0.926 | 0.928 | {'C': 0.1} | ['Age', 'oxLDL-C', 'ALB', 'LDL-C', 'HDL-C', 'CK-MB', 'Glu', 'CHO', 'eGFR', 'AG'] |
| 8 | 0.905 | 0.930 | {'C': 0.1} | ['Age', 'oxLDL-C', 'ALB', 'LDL-C', 'HDL-C', 'CK-MB', 'Glu', 'CHO', 'eGFR', 'AG'] |
| 9 | 0.943 | 0.921 | {'C': 0.1} | ['Age', 'oxLDL-C', 'ALB', 'LDL-C', 'HDL-C', 'CK-MB', 'Glu', 'CHO', 'eGFR', 'NT-proBNP'] |
| 10 | 0.925 | 0.923 | {'C': 0.1} | ['Age', 'oxLDL-C', 'ALB', 'LDL-C', 'HDL-C', 'CK-MB', 'Glu', 'CHO', 'eGFR', 'NT-proBNP'] |

Abbreviations: RFE, recursive feature elimination; AUC, area under the receiver operating characteristic curve; oxLDL-C, oxidized low-density lipoprotein cholesterol; ALB, albumin; LDL-C, low-density lipoprotein cholesterol; HDL-C, high-density lipoprotein cholesterol; CK-MB, creatine kinase-MB; Glu, glucose; CHO, total cholesterol; eGFR, estimated glomerular filtration rate; NT-proBNP, N-terminal pro-brain natriuretic peptide; AG, anion gap; CV, coefficient of variation.

| **Supplementary Table S7. Hyperparameter Configurations of the Final Machine Learning Models** | |  |
| --- | --- | --- |
|  |  |  |
| Machine learning models | parameter configuration |  |
| DT | {'ccp_alpha': 0.0, 'max_depth': 5, 'max_features': None, 'min_samples_split': 20} |  |
| RF | n_estimators = 450 , max_features = 2 |  |
| XGBoost | {'colsample_bytree': 0.7, 'learning_rate': 0.05, 'max_depth': 3, 'min_child_weight': 5, 'n_estimators': 200, 'reg_lambda': 1, 'subsample': 0.7} |  |
| LightGBM | {'colsample_bytree': 0.8, 'learning_rate': 0.1, 'n_estimators': 100, 'num_leaves': 31, 'subsample': 0.6} |  |
| SVM | {'C': 0.1, 'degree': 2, 'gamma': 'scale', 'kernel': 'linear'} |  |
| ANN | {'activation': 'logistic', 'hidden_layer_sizes': (50, 50, 50)} |  |

Abbreviations: DT, Decision Tree; RF, Random Forest; XGBoost, Extreme Gradient Boosting; LightGBM, Light Gradient Boosting Machine; SVM, Support Vector Machine; ANN, Artificial Neural Network.

**Supplementary Table S8. Pairwise DeLong Test for AUC Comparisons Among Seven Models**

| Model | Logistic | Decision Tree | Random Forest | XGBoost | LightGBM | SVM | ANN |
| --- | --- | --- | --- | --- | --- | --- | --- |
| Training Set |  |  |  |  |  |  |  |
| Logistic | 1.000 | ＜0.001 | ＜0.001 | ＜0.001 | ＜0.001 | 0.311 | ＜0.001 |
| Decision Tree | ＜0.001 | 1.000 | ＜0.001 | ＜0.001 | ＜0.001 | ＜0.001 | 0.035 |
| Random Forest | ＜0.001 | ＜0.001 | 1.000 | ＜0.001 | 0.986 | ＜0.001 | ＜0.001 |
| XGBoost | ＜0.001 | ＜0.001 | ＜0.001 | 1.000 | ＜0.001 | ＜0.001 | ＜0.001 |
| LightGBM | ＜0.001 | ＜0.001 | 0.986 | ＜0.001 | 1.000 | ＜0.001 | ＜0.001 |
| SVM | 0.311 | ＜0.001 | ＜0.001 | ＜0.001 | ＜0.001 | 1.000 | ＜0.001 |
| ANN | ＜0.001 | 0.035 | ＜0.001 | ＜0.001 | ＜0.001 | ＜0.001 | 1.000 |
| Validation set |  |  |  |  |  |  |  |
| Logistic | 1.000 | 0.084 | 0.008 | 0.001 | 0.553 | 0.993 | 0.206 |
| Decision Tree | 0.084 | 1.000 | ＜0.001 | ＜0.001 | 0.010 | 0.082 | 0.010 |
| Random Forest | 0.008 | ＜0.001 | 1.000 | 0.132 | 0.001 | 0.009 | 0.067 |
| XGBoost | 0.001 | ＜0.001 | 0.132 | 1.000 | ＜0.001 | 0.001 | 0.008 |
| LightGBM | 0.553 | 0.010 | 0.001 | ＜0.001 | 1.000 | 0.560 | 0.759 |
| SVM | 0.993 | 0.082 | 0.009 | 0.001 | 0.560 | 1.000 | 0.198 |
| ANN | 0.206 | 0.010 | 0.067 | 0.008 | 0.759 | 0.198 | 1.000 |

Abbreviations: DT, Decision Tree; RF, Random Forest; XGBoost, Extreme Gradient Boosting; LightGBM, Light Gradient Boosting Machine; SVM, Support Vector Machine; ANN, Artificial Neural Network.

Note: P values are derived from DeLong test. Values in bold indicate the key comparison between Logistic Regression and XGBoost in the validation set.

**Supplementary Figure 1. Correlation heatmap of the selected variables**
